# Supplementary material for: Association of early childhood abdominal circumference and weight gain with blood pressure at 36 months of age: secondary analysis of data from a prospective cohort study
Source: BMJ Open. 2014 Jul 3;4(7):e005412. doi: 10.1136/bmjopen-2014-005412 (PMC4091398; doi:10.1136/bmjopen-2014-005412)
Supplement: Author's manuscript [file bmjopen-2014-005412.draft_revisions.pdf]

Influence of early childhood abdominal circumference and weight gain on blood pressure at 36 months of age: secondary analysis of data from a prospective cohort study

Journal:	*BMJ Open*
Manuscript ID:	bmjopen-2014-005412
Article Type:	Research
Date Submitted by the Author:	06-Apr-2014
Complete List of Authors:	Nowson, Caryl; Deakin University, Centre for Physical Activity and Nutrition Research<br>Crozier, S; University of Southampton & Southampton General Hospital, MRC Lifecourse Epidemiology Unit<br>Robinson, S; University of Southampton & Southampton General Hospital, MRC Lifecourse Epidemiology Unit<br>Godfrey, Keith; University of Southampton & Southampton General Hospital, MRC Lifecourse Epidemiology Unit; University of Southampton & University Hospital Southampton NHS Foundation Trust, NIHR Southampton Biomedical Research Centre<br>Lawrence, Wendy; University of Southampton & Southampton General Hospital, MRC Lifecourse Epidemiology Unit<br>Law, Catherine; University College London, UCL Institute of Child Health<br>Cooper, Cyrus; University of Southampton & Southampton General Hospital, MRC Lifecourse Epidemiology Unit<br>Inskip, Hazel; University of Southampton & Southampton General Hospital, MRC Lifecourse Epidemiology Unit
**Primary Subject Heading**:	Paediatrics
Secondary Subject Heading:	Epidemiology, Cardiovascular medicine, Nutrition and metabolism, Occupational and environmental medicine, Public health
Keywords:	Hypertension < CARDIOLOGY, Cardiac Epidemiology < CARDIOLOGY, Paediatric cardiology < CARDIOLOGY, EPIDEMIOLOGY, Community child health < PAEDIATRICS, PREVENTIVE MEDICINE

Influence of early childhood abdominal circumference and weight gain on blood pressure at 36 months of age: secondary analysis of data from a prospective cohort study

Caryl A Nowson[1], Sarah R Crozier[2], Siân M Robinson[2], Keith M Godfrey[2,3], Wendy T Lawrence[2], Catherine M Law[4], Cyrus Cooper[2] and Hazel M Inskip[2]

[1]Centre of Physical Activity and Nutrition Research, School of Exercise and Nutrition Sciences, Deakin University, Burwood, Victoria, Australia

[2]MRC Lifecourse Epidemiology Unit, University of Southampton & Southampton General Hospital, Southampton, UK

[3]NIHR Southampton Biomedical Research Centre, University of Southampton & University Hospital Southampton NHS Foundation Trust, Southampton, UK

[4]MRC Centre of Epidemiology for Child Health/Centre for Paediatric Epidemiology and Biostatistics, UCL Institute of Child Health, London, UK

Corresponding author:

Professor Caryl A. Nowson, Centre for Physical Activity and Nutrition Research, Deakin University, Locked Bag 200000, Geelong, VIC. 3220 caryl.nowson@deakin.edu.au , Tel: +61 3 5247 9245, Fax: +61 3 5227 8376

Key words: paediatrics, growth and development, blood pressure, body weight, abdominal circumference

Abbreviations: BMI: body mass index; CI: confidence interval; m: months, SWS: Southampton Women's Survey

Wordcount: 2,956

For peer review only

ABSTRACT

Objectives: To assess if changes in measures of fat distribution and body size during early life are associated with blood pressure at 36 months of age.

Design: Analysis of data collected from a prospective cohort study.

Setting: Community-based investigation in Southampton, UK.

Participants: 761 children with valid blood pressure measurements, born to women participating in the Southampton Women's Survey.

Primary and secondary outcome measures: Anthropometric measurements were collected at 0, 6, 12, 24 and 36 months (mo) and conditional changes between the time-points calculated. Blood pressure was measured at 36 mo. Factors possibly influencing the blood pressure were assessed using linear regression. All independent variables of interest and confounding variables were included in stepwise multiple regression to identify the model that best predicted blood pressure at 36mo.

Results: Greater conditional gains in abdominal circumference between 0–6 and 24–36mo were associated with higher systolic and diastolic blood pressures at 36mo (P<0.001). Subscapular skinfold and height gains were weakly associated with higher blood pressures, while greater weight gains between 0–6, 12–24 and 24–36mo were more strongly associated, but the dominant influences were abdominal circumference gains. Systolic blood pressure increased by 0.87 mmHg (95% CI: 0.02,1.71) per standard deviation score (SDS) increase in abdominal circumference between 0-6mo, and 1.64 mmHg (1.02, 2.27) between 24–36mo; diastolic pressure increased by 0.98 mmHg (0.51, 1.44) per SDS increase between 0–6mo, and 0.90 mmHg (0.43, 1.38) between 24–36mo.

Conclusions: Conditional gains in abdominal circumference, particularly within six months of birth and in the year preceding measurement, were more positively associated with blood pressure at

For peer review only - http://bmjopen.bmj.com/site/about/guidelines.xhtml

36mo than gains in other anthropometric measures. Above-average abdominal circumference gains in early childhood may contribute to adult hypertension and increased cardiovascular disease risk.

Strengths and Limitations of this Study

- This is one of few studies that have investigated detailed anthropometric changes in relation to blood pressure in early age and examined conditional changes between different age-points.
- Key confounding risk factors were adjusted for in the models, including maternal education attainment and smoking during pregnancy.
- A large number of children from a cross-section of socioeconomic backgrounds were included in the study.
- We were not able to include all the children born in the course of the cohort study as blood pressure measurements were not available for all children, but the study sample was found to be similar to the larger group at 36 months of age.
- Abdominal girth at this young age may only represent a gross measure of central fat deposition and differences between individuals may represent genetically/prenatally-determined differences in physique.

For peer review only

For peer review only - http://bmjopen.bmj.com/site/about/guidelines.xhtml

INTRODUCTION

Low birth weight and rapid postnatal weight gain have been linked to increased risk of cardiovascular disease,[1] obesity, and the metabolic syndrome - including hypertension[2] and insulin resistance[3] - later in life. Accelerated weight gain, characterised by above-average velocities of skeletal and non-skeletal postnatal growth, has been associated with higher blood pressure in childhood.[4] Low birth weight predicts blood pressure in later life,[5] but it is not clear how much this association can be attributed to low birth weight independently of accelerated postnatal weight gain, as infants who are born small for gestational age tend to gain weight more rapidly during the early postnatal period.[6]

It is thought that there may be critical periods at specific time-points early in life when accelerated growth predisposes to hypertension later in life.[7-10] Furthermore, rapid increase in weight-for-length in the first 6 months has been associated with higher systolic blood pressure in 3-year-olds.[11] Few studies have assessed indicators of body fat distribution in infants and young children. Body fat distribution has been associated with risk factor scores for cardiovascular risk in young children[12] and postnatal rapid weight gain has been linked to deposition of fat centrally in children at 5 years.[6]

Therefore, insight into whether postnatal alterations in body composition influence blood pressure in early childhood is relevant to the development of preventative strategies to reduce the risk of cardiovascular disease in later life. Our aim was to assess how gains in adiposity, fat distribution and body size between birth, 6, 12, 24 and 36 months relate to the blood pressure of children at 36 months.

For peer review only - http://bmjopen.bmj.com/site/about/guidelines.xhtml

METHODS

Study sample: the Southampton Women's Survey (SWS)

The SWS is a large prospective cohort study which commenced in 1998.[13] A total of 12,583 non-pregnant women aged 20 to 34 years were recruited to the study. Detailed information on diet and socio-demographic factors was collected and children born to SWS women were assessed at birth and then followed up at home by trained research nurses. The SWS was approved by the Southampton and South West Hampshire Local Research Ethics Committee, and participants gave written informed consent. The research conformed to the principles embodied in the Declaration of Helsinki.

There were 1,981 singleton live births to women in the SWS by the end of 2003. After exclusion of infants with major congenital abnormalities (n=2) and neonatal deaths (n=6), 1,973 SWS infants remained for postnatal follow-up.

Maternal and child data

When each child was 24 months old, the occupations of its mother and her partner were recorded and the highest-ranking of these used to define the child's social class. The social class scale was: Professional (I), Management and technical (II), Skilled non-manual (IIIN), Skilled manual (IIIM), Partly skilled (IV), and Unskilled (V). For 10 children whose parental occupations were missing at this time, employment status recorded during early pregnancy was used. Educational attainment of the mother recorded before pregnancy was defined in six groups, from 'none' to 'degree or above'.

For peer review only - http://bmjopen.bmj.com/site/about/guidelines.xhtml

Body composition and blood pressure assessment

Anthropometric measurements were taken by trained researchers at birth, 6mo, 12mo, 24mo and 36mo. Apart from those at birth, all measurements were taken in the children's homes. Infant crown-heel length was measured with a neonatometer (CMS Ltd, London, United Kingdom). Child height was measured with a portable stadiometer (Leicester height measurer; CMS Ltd). Skinfold thicknesses were measured using Holtain skinfold callipers (Holtain Ltd) at specified sites, and abdominal circumference measured at the end of expiration using a blank tape measured against a fixed scale. Strict monitoring of the nurses' measurement techniques was performed by the senior research nurse and regular inter-observer variation studies were conducted.

Blood pressure was measured using a Critikon DINAMAP 1846 SX automated blood pressure device [14] with the child seated. Three measurements were recorded and the average of the last two used in the analysis. Due to limited equipment availability, blood pressure measurements were only available for approximately 47% of the SWS children.

Statistical analysis

Regression coefficients (β), with associated 95 % confidence intervals (CI), were used to assess the strength of association between body size indicators (body weight, length/height, abdominal circumference and subscapular skinfold thickness). Z-scores were calculated for body weight and length/height using the 1990 British growth references for time points 6mo, 12mo, 24mo and 36mo.[15] Z-scores for abdominal circumference and subscapular skinfolds were calculated internally using the SWS sample and were adjusted for gender, current age and gestational age. Conditional growth was derived from the residuals resulting from regression of the z-score for the measurement at a specific time point on the z-scores for measurements at all preceding ages. For example, the

For peer review only - http://bmjopen.bmj.com/site/about/guidelines.xhtml

dependent variable 'conditional gain in body weight from 12mo to 24mo' was derived as the residual of the regression of body weight z-score at 24mo on the z-scores for body weight at 12mo, 6mo and birth.

Factors reported to be associated with blood pressure were assessed using linear regression, including:, age, social class, maternal education attainment, smoking in late pregnancy (an indicator of smoking throughout pregnancy) and crying of the child during blood pressure measurement. Factors that were univariately associated with blood pressure at age 36mo were retained for inclusion in regression models that explored the relationship between blood pressure and body composition or fat distribution, but were subsequently dropped if they did not remain significant at the 5% level in the final models.

Multiple regression analysis was performed by entering all independent variables of interest into the model, in addition to the confounding variables. A stepwise multiple regression analysis was used to identify the model that best predicted blood pressure at 36mo. Statistical analyses were performed using SPSS PASW Statistics Release 18 (IBM SPSS, IBM Corp, New York), and Stata 12.0 (StataCorp, Texas, USA).

RESULTS

At 36mo of age, 1,640 children (83% of the cohort) were followed-up. Birth weights and 36mo blood pressure measurements were then available for 773 infants. Seven children with missing height and weight data at age 36mo and five with systolic pressures more than 3 standard deviations from the mean were excluded, leaving 761 in the analysis. Children in this study were similar to the larger population sample of SWS children at 36mo (Table 1). Owing to a relative

For peer review only - http://bmjopen.bmj.com/site/about/guidelines.xhtml

For peer review only

unavailability of blood pressure machines during later fieldwork, children included in the analyses were more likely to have been studied earlier in the study. The full ranges of social classes and educational levels were represented in the analysis sample, although the ‘Professional/Management & technical’ social class accounted for around 40% of the population, and just over half the mothers had completed higher school/post-school qualifications.

For peer review only

For peer review only - http://bmjopen.bmj.com/site/about/guidelines.xhtml

Table 1. Maternal and infant characteristics of the Southampton Women's Survey study group

	With BP measurement (n=761)		Without BP measurement (n=879)	
**Mothers**	Mean	SD	Mean	SD
Maternal age at birth of the child	29.7	3.7	30.6	3.8
Weight (kg)[1]	67.3	13.9	68.3	13.9
Height (m)	163.0	6.5	163.4	6.3
Maternal BMI (pre-pregnancy) (kg/m^2)[1]	25.3	4.8	25.5	4.8
	Percent		Percent	
Smoking in pregnancy	16.0		12.0	
Social class				
Professional/Management & technical (I/II)	40.2		40.0	
Skilled manual/non-manual (III)	43.4		45.9	
Partly skilled/unskilled (IV/V)	16.4		14.1	
Educational attainment				
Compulsory education to age 16 years	44.9		38.9	
Post compulsory education	55.1		65.1	
**Infants**				
Gender - male, n (%)	397(52)		473 (54)	
	Mean	SD	Mean	SD
Age in years at 36mo visit	3.1	0.1	3.1	0.1
Weight (kg) at 36mo visit	15.0	1.9	15.1	1.8
Height (cm) at 36mo visit	95.7	3.7	96.0	3.6

For peer review only

Abdominal circumference (cm) at 36mo visit	51.2	3.2	51.3	3.1
Subscapular skinfold (mm) at 36mo visit	6.6	1.8	6.5	1.8
Systolic blood pressure (mmHg) at 36mo visit	93.8	8.3		
Diastolic blood pressure (mmHg) at 36mo visit	58.1	6.3		
Birth weight (kg)	3.4	0.6	3.5	0.5
Birth length (cm)	49.8	2.1	50.0	2.0

BP: blood pressure, SD: standard deviation, BMI: body mass index

[1]based on reported weight 3 months prior to pregnancy

For peer review only

For peer review only - http://bmjopen.bmj.com/site/about/guidelines.xhtml

The greatest relative and absolute increases in body weight, height/length, abdominal circumference and subscapular skinfold thickness occurred between birth and 6mo (Table 2), with the other age intervals (6-12mo, 12-24mo and 24-36mo) indicating smaller positive increments for body weight and height. Mean values of height and weight were comparable to the 50th percentile.[15] For subscapular skinfold, average changes between later ages from 6m were negative, as was the average change in abdominal circumference between 24 and 36mo. During this final age period, abdominal circumference increased in 39% of children but decreased in 60%.

Confounding variables

Age, gender and social class were not associated with blood pressure, but the 45 infants who cried during measurement had higher systolic (P=0.004) and diastolic pressures (P=0.001). Smoking in late pregnancy was associated with higher systolic (P=0.067) and diastolic pressures (p=0.005). Lower educational attainment (P=0.024) was associated with higher diastolic pressure. However, in the final models, smoking in late pregnancy was not significantly related to either systolic or diastolic pressure, so was dropped.

For peer review only

For peer review only - http://bmjopen.bmj.com/site/about/guidelines.xhtml

Table 2. Body composition at 0, 6, 12, 24, and 36 months and incremental changes

				Absolute change		% change[1]	
	Mean	SD		Mean	SD	Mean	SD
N=712							
Birth weight (kg)	3.4	0.6	*Δ wt (kg): 0 - 36mo*	*11.6*	*1.8*	*349.7*	*92.7*
Weight (kg) 6mo	7.9	1.0	Δ wt (kg): 0 - 6mo	4.4	0.9	134.4	43.6
Weight (kg) 12mo	10.0	1.2	Δ wt (kg): 6 - 12mo	2.1	0.6	27.2	8.2
Weight (kg) 24mo	12.5	1.5	Δ wt (kg):12 - 24mo	2.6	0.9	26.0	8.5
Weight (kg) 36mo	15.0	1.9	Δ wt (kg): 24 - 36mo	2.5	0.9	20.1	7.0
N=666							
Birth length (cm)	49.8	2.1	*Δ ht (cm): 0 - 36mo*	*46*	*3.2*	*92.7*	*8.3*
Height (cm) 6mo	67.2	2.5	Δ ht (cm): 0 - 6mo	17.4	2.1	35.1	5.0
Height (cm) 12mo	75.6	2.7	Δ ht (cm): 6 - 12mo	8.4	1.8	12.6	2.8
Height (cm) 24mo	86.3	3.1	Δ ht (cm): 12 - 24mo	10.8	1.9	14.3	2.5
Height (cm) 36mo	95.8	3.6	Δ ht (cm): 24 - 36mo	9.5	1.6	11.0	1.9

For peer review only

N=682							
Abdominal circum.[2](cm) 0mo	31.5	2.2	*Δ circum. (cm): 0 - 36mo*	*19.8*	*3.1*	*63.3*	*12.3*
Abdominal circum. (cm) 6mo	47.4	3.2	Δ circum. (cm): 0 - 6mo	15.9	3.3	50.9	12.3
Abdominal circum.(cm) 12mo	49.6	3.2	Δ circum. (cm): 6 -12mo	2.2	2.6	4.7	5.7
Abdominal circum. (cm) 24mo	51.9	3.4	Δ circum. (cm): 12 - 24mo	2.3	2.9	4.8	6.0
Abdominal circum.(cm) 36mo	51.3	3.1	Δ circum. (cm): 24 - 36mo	-0.6	2.5	-1.0	4.7
N=645							
Subscapular skinfold (mm) 0mo	5.0	1.0	*Δ subscap[3].(mm): 0 - 36mo*	*1.7*	*1.9*	*37.2*	*42.4*
Subscapular skinfold (mm) 6mo	7.4	1.6	Δ subscap. (mm): 0 - 6mo	2.4	1.7	52.7	41.7
Subscapular skinfold (mm) 12mo	7.2	1.6	Δ subscap. (mm): 6- 12mo	-0.2	1.5	-1.8	19.3
Subscapular skinfold (mm) 24mo	6.6	1.6	Δ subscap. (mm): 12 -24mo	-0.6	1.4	-7.3	19.3
Subscapular skinfold (mm) 36mo	6.7	1.9	Δ subscap. (mm): 24 -36mo	0.1	1.4	3.6	20.1

SD: standard deviation

[1] calculated from previous time interval [2]abdominal circumference [3]subscapular skinfold thickness

For peer review only

Predictors of blood pressure at 36 months

Initially, the four anthropometric measurements were considered separately (Table 3). Each model contained the measurement at birth and the conditional changes in the measure over the four age periods (0-6mo, 6-12mo, 12-24mo, and 24-36mo), along with the confounding factors that contributed significantly to the regression analysis; the slope represents the change in blood pressure (mmHg) per SD change in growth measurement. In all four models, the measurements at birth were not associated with blood pressure, independently of measurements of postnatal growth. The model for abdominal circumference explained more of the variance in blood pressure (9.0% and 7.7%, respectively, for systolic and diastolic blood pressure) (Figure 1) than the models for the other three measures, with the model for weight coming a close second (6.8% and 5.3% of the variance explained for systolic and diastolic blood pressure). The change in abdominal circumference in the final age period (24-36mo) was related most strongly to blood pressure (both systolic and diastolic), but change during the first six months of life was also an important predictor. Weight change between birth and 6mo was related to blood pressure at 36mo, but weight change between 12 and 24mo also appeared to influence both systolic and diastolic blood pressure. Neither height nor subscapular skinfold thickness changes were related to blood pressure as strongly as abdominal circumference or weight changes, although for diastolic blood pressure there was a robust association with height change between 12 and 24mo. The effect sizes for the significant associations of body fat distribution and body weight were such that a 1 SDS increase in the measurement between the two ages under consideration was associated with an increase of around 1-2 mmHg in systolic blood pressure and approximately 1 mmHg in diastolic pressure

For peer review only - http://bmjopen.bmj.com/site/about/guidelines.xhtml

Table 3. Multiple regression models: associations between conditional gain (z-scores) in body composition measures and blood pressure[1]

	Systolic Pressure[2]					Diastolic Pressure[3]				
	Adj.R^2	β	95% CI		P- value	Adj.R^2	β	95% CI		P-value
**Model 1 – Weight[4] (N=712)**	0.068					.053				
Birth weight z-score		0.23	-0.41	0.88	0.5		0.04	-0.44	0.52	0.9
Weight: 0-6mo[5]		1.41	0.82	1.99	<0.001		0.82	0.38	1.26	<0.001
Weight: 6-12mo[5]		0.48	-0.11	1.07	0.1		0.34	-0.11	0.78	0.1
Weight: 12-24mo[5]		1.17	0.58	1.76	<0.001		0.73	0.29	1.18	0.001
Weight: 24-36mo[5]		1.03	0.44	1.62	0.001		0.43	-0.02	0.87	0.1
**Model 2 – Length/height[4] (N=666)**	0.029					.038				
Birth length z-score		0.46	-0.29	1.20	0.2		-0.02	-0.56	0.53	0.95
Height: 0-6mo[5]		0.88	0.26	1.50	0.006		0.39	-0.07	0.85	0.1
Height: 6-12mo[5]		0.67	0.04	1.30	0.04		0.27	-0.20	0.73	0.3
Height: 12-24mo[5]		0.73	0.10	1.36	0.02		0.74	0.27	1.21	<0.001
Height: 24-36mo[5]		0.33	-0.30	0.96	0.3		0.18	-0.28	0.65	0.4

For peer review only

**Model 3 – Abdominal circumference[6] (N=682)**	.090					.077				
Birth abdominal circumference z-score		0.27	-0.32	0.87	0.4		-0.29	-0.74	0.16	0.2
Abdominal circumference[5]: 0-6mo		1.56	0.95	2.17	<0.001		1.06	0.60	1.52	<0.001
Abdominal circumerence[5]: 6-12mo		-0.03	-0.63	0.57	0.9		-0.21	-0.67	0.25	0.4
Abdominal circumerence[5]: 12-24mo		0.62	0.02	1.22	0.04		0.42	-0.04	0.87	0.07
Abdominal circumerence[5]: 24-36mo		1.92	1.32	2.51	<0.001		0.97	0.52	1.43	<0.001
**Model 4 – Subscapular skinfold thickness[6] (N=645)**	0.02					.033				
Birth subscapular skinfold		-0.08	-0.74	0.58	0.8		-0.30	-0.79	0.19	0.2
Subscapular skinfold[5]: 0-6mo		0.42	-0.23	1.07	0.2		0.16	-0.33	0.64	0.5
Subscapular skinfold[5]: 6 - 12mo		0.93	0.27	1.58	0.01		0.76	0.28	1.25	0.002
Subscapular skinfold[5]: 12 - 24mo		0.55	-0.10	1.19	0.1		0.05	-0.42	0.53	0.8
Subscapular skinfold[5]: 24 - 36mo		0.45	-0.20	1.10	0.2		0.25	-0.22	0.73	0.3

CI: confidence interval

[1]residuals derived from regression model with the specified z-score as the independent variable, [2]crying included in the model , [3]crying and maternal educational level included in the model, [4]z-scores derived from percentile curve growth charts UK [15], [5]adjusted for measurements at all preceding time points, [6]z-scores derived internally from Southampton Women's Survey data.

For peer review only

The results of the final regression models are presented in Table 4. Both abdominal circumference and weight changes were associated with systolic blood pressure. Not surprisingly, weight gains between birth and 6mo and between 12 and 24mo were associated with abdominal circumference change during these same age periods (r = 0.7 and 0.5 respectively). There was considerable variability in the gains of abdominal mass between 24 to 36mo, where 39% of children experienced an increase in abdominal circumference. Abdominal circumference change in the age period leading up to the blood pressure measurement was the key influence on blood pressure, with a 1 SDS change in the circumference being associated with a 1.64 mmHg increase in systolic blood pressure. Early abdominal circumference and weight changes between birth and 6mo were also significantly associated with systolic blood pressure. The final model for diastolic blood pressure was slightly different, though key predictors were still abdominal circumference change in the earliest and latest age periods (0-6mo and 24-36mo) with a 1 SDS increase in abdominal circumference change associated with approximately 1 mmHg higher diastolic blood pressure. After the abdominal circumference changes were included in the model, changes in weight no longer appeared to influence blood pressure, but height change between 12 and 24mo was retained in the model. The addition of the variables 'ever breast-fed' or 'duration of breast feeding' to the final models did not affect the relationships between blood pressure and body composition measures.

For peer review only

For peer review only - http://bmjopen.bmj.com/site/about/guidelines.xhtml

Table 4. Multivariate regression model of best fit: conditional gain (z-scores)[1] in body size and fat distribution associations with blood pressure at 36 months

Systolic Pressure[2]

N=668	Adj.R^2	β	95% CI		P-value
	.098				
Abdominal circumference[3,4]: 0-6mo		0.87	0.02	1.71	0.04
Abdominal circumference[3,4]: 24-36mo		1.64	1.02	2.27	<0.0001
Weight: 0-6mo[3,5]		0.94	0.06	1.82	0.04
Weight: 12-24mo[3,5]		0.77	0.16	1.39	0.01

Diastolic Pressure[6]

N=641	Adj.R^2	β	95% CI		P-value
	.074				
Abdominal circumference[3,4]: 0-6mo		0.98	0.51	1.44	<0.001
Abdominal circumference[3,4]: 24-36mo		0.90	0.43	1.38	<0.001
Height: 12-24mo[3,5]		0.60	0.12	1.07	.01

CI: confidence interval

[1]residuals derived from regression model with the specified z-score as the independent variable, [2]crying included in the model, [3]adjusted for all the preceding time intervals (0-6mo, 6-12mo, 12-24mo, 24-36mo), [4]Z-scores based on sample population data from Southampton Women's Survey, [5]z-scores derived from percentile curve growth charts UK [15], [6]crying and maternal educational level included in the model

For peer review only

For peer review only - http://bmjopen.bmj.com/site/about/guidelines.xhtml

DISCUSSION

Main findings

Our data indicate that conditional gains in abdominal circumference between birth and 6 months, as well as in the 12 months prior to blood pressure measurement, are significant predictors of both systolic and diastolic blood pressure at 36 months of age. Changes in weight were also associated with blood pressure, particularly systolic pressure, but to a lesser extent. A one-SDS z-score gain in abdominal circumference between 24 and 36mo was associated with a 1.64 mmHg increase in systolic blood and a one-SDS increase between birth and 6mo with 0.87 mmHg.

There was considerable variability in the gains of abdominal mass at the time interval most predictive of blood pressure (24 to 36mo), with 39% of children experiencing an increase, suggesting the impact of environmental factors. It is possible that dietary intake and/or physical activity levels during this early period could alter abdominal circumference gain early in life, or it may be related to differences in growth trajectories,[16] which predict later obesity. Given that abdominal circumference in the majority of children decreases between the ages of 24 and 36mo, increases seen in this time period might be an early marker for problems in later life, particularly if the effect of abdominal circumference change on blood pressure amplifies with age.

It is of note that subscapular skinfold thickness change, an indicator of subcutaneous fat deposition, had much weaker associations with blood pressure than abdominal circumference, which is an indicator of abdominal or visceral fat deposition. The finding that abdominal circumference gain between 0-6mo was more strongly associated with blood pressure at age 36mo than gains during 6-12mo and 12-24mo could reflect the proposed critical period for childhood adiposity in the first two months of life,[17] or could reflect gains in adiposity associated with an adverse intrauterine environment in late gestation.

For peer review only - http://bmjopen.bmj.com/site/about/guidelines.xhtml

Strengths and weaknesses

As summarised earlier, a strength of this study is the large sample size drawn from the general population. In addition, we have assessed conditional growth, accounting for measurements at previous ages (thus ensuring independence of growth summaries between age periods), and have adjusted for relevant confounding factors. Few studies have collected such detailed anthropometric measurements with the methodological rigor to provide indicators of fat distribution in the early postnatal period, encompassing the transition from milk-only to a mixed diet at the age of 36 months.

A limitation of the study is the inability to measure blood pressure in all children in the cohort, although there do not appear to be noteworthy differences between those who were measured and the remainder.

Comparison with other studies

Central adiposity and a large waist circumference have been associated with higher blood pressure in adults, but there are few reports for children. In one study, children with accelerated weight gain in the first two years of life were fatter and had more central fat distribution at five years of age than other children.[6] However, the relationship of central fat deposition specifically to blood pressure, which is only one component of the metabolic syndrome, is not clear in children. Rapid weight gain during infancy (0–6mo) but not during early childhood (3–6 years) was associated with a higher metabolic risk score at age 17 years, but the association between waist circumference and blood pressure was not statistically significant in that study.[18] Waist-for-height ratio in school children has not been found to confer additional discriminative power to BMI in some studies,[19, 20] but recently weight and weight-for-height changes through infancy and childhood have been associated with blood pressure at age 10 years[21] and at age 9.1 and 15.5 years.[22] These studies did not consider the periods 0-6mo and 6-12mo separately, nor examine change in abdominal circumference.

For peer review only - http://bmjopen.bmj.com/site/about/guidelines.xhtml

However, similar to both reports, we found that weight changes at ages closest to those at which the blood pressure measurement was made were among the strongest predictors.

There is considerable debate as to whether there are specific age-periods early in life during which weight gain and/or fat deposition are more influential in predicting cardiovascular risk. Our findings are similar to a report that weight gain between 24 and 36mo was positively associated with systolic blood pressure.[23] The mean blood pressure values in our study of 94/58 mmHg are similar to those reported (92/58 mmHg),[23] as are the correlations between systolic blood pressure and current weight and height at 36mo. Similarly, we also did not find an inverse association between birth weight and blood pressure. However, in contrast to the other findings that blood pressure was related to weight change between 24 and 36mo but not earlier, we found that conditional gains in body weight between all age periods were positively associated with systolic blood pressure, except for the period between 6 to 12mo. The differences in results may be related to our adjustment for all previous time intervals when we determined conditional growth.

Our data support a developmental contribution to the origin of elevated blood pressure in childhood, and therefore potentially in later adulthood. Indeed, we have previously demonstrated that higher fetal liver blood flow is strongly correlated with greater fat mass at birth and at 4 years, indicating that influences on body composition operate very early in life.[24] Our data show that changes in abdominal circumference during the first six months and in the third year of life are associated with systolic and diastolic blood pressure. This suggests that the tracking of blood pressure, evident by later childhood, might be a consequence of under-supply of conditionally essential nutrients associated with elevated fetal liver blood flow and prioritisation of fat deposition in the infant.

It is not clear whether postnatal interventions would be effective in limiting growth in abdominal circumference, and any interventions to limit abdominal circumference gains would likely also reduce the rate of growth. However, there is accumulating evidence that accelerated early growth has long-term adverse

For peer review only - http://bmjopen.bmj.com/site/about/guidelines.xhtml

physiological effects in later life, increasing cardiovascular risk factors, including high blood pressure.[25] Promotion of lifestyle practices, such as exclusive breast feeding up to 6 months, later introduction of solid foods and maximising opportunities for safe physical activity, could contribute to a reduction in accelerated growth early in life and be one pathway that results in a reduction in cardiovascular disease later in life.

Conclusion

We have demonstrated that although conditional gains in body weight and height during specific age periods are associated with systolic and diastolic blood pressure, as previously recognised, conditional gains in abdominal circumference (associated with central fat deposition) are the strongest predictors of blood pressure at 36 months of age. This is especially true for the first 6 months of life and between 24 and 36 months (i.e., the year prior to blood pressure measurement). Therefore, central deposition of fat in early childhood, indicated by abdominal circumference, may contribute to an increased risk of developing hypertension later in life. Follow-up blood pressure measurements for the study group may enable confirmation of this at a later date.

For peer review only - http://bmjopen.bmj.com/site/about/guidelines.xhtml

Figure 1

Association between change in abdominal circumference (residuals derived from regressing the z-score at each specific age on the z-score at all preceding ages) as the independent variable and blood pressure at 36 months (mmHg). Z-scores are based on data from the Southampton Women's Survey, adjusted for gender, current age and gestational age.

For peer review only

For peer review only - http://bmjopen.bmj.com/site/about/guidelines.xhtml

Acknowledgements

The Southampton Women's Survey Study Group includes Patsy Coakley, Vanessa Cox, Julia Hammond, Tina Horsfall and the Research Nurses who collected and processed the data for the Survey. The authors are very grateful to the families who participated in the research.

Competing Interests

None

Funding

This work was supported by grants from the Medical Research Council, British Heart Foundation, Arthritis Research UK, National Institute for Health Research (NIHR) Southampton Biomedical Research Centre, University of Southampton and University Hospital Southampton NHS Foundation Trust, and NIHR Musculoskeletal Biomedical Research Unit, University of Oxford. The work was also financially supported in part by the Commission of the European Community, specifically the RTD Programme "Quality of Life and Management of Living Resources", within the 7th Framework Programme, research grant no. FP7/2007-13 (Early Nutrition Project). This manuscript does not necessarily reflect the views of the funders and in no way anticipates the future policy in this area. KMG is supported by the National Institute for Health Research through the NIHR Southampton Biomedical Research Centre.

Author contributions

Caryl Nowson conceptualised the study, performed the analysis, contributed to the interpretation and drafted the initial manuscript. Sarah Crozier assisted with the analysis and contributed to the interpretation. Siân Robinson contributed to interpretation. Keith Godfrey, Wendy Lawrence, Catherine Law and Cyrus Cooper

For peer review only - http://bmjopen.bmj.com/site/about/guidelines.xhtml

reviewed and revised the manuscript. Hazel Inskip conceptualised the study and designed the analysis plan, performed analysis, contributed to the interpretation and revised the manuscript. All authors approved the final manuscript as submitted.

Data sharing

No additional data available.

For peer review only

REFERENCES

1 Ioannou C, Javaid MK, Mahon P*, et al.* The effect of maternal vitamin D concentration on fetal bone. *J Clin Endocrinol Metab* 2012;97:E2070-7.

2 Law CM, Shiell AW, Newsome CA*, et al.* Fetal, infant, and childhood growth and adult blood pressure: a longitudinal study from birth to 22 years of age. *Circulation* 2002;105:1088-92.

3 Noakes PS, Vlachava M, Kremmyda LS*, et al.* Increased intake of oily fish in pregnancy: effects on neonatal immune responses and on clinical outcomes in infants at 6 mo. *Am J Clin Nutr* 2012;95:395-404.

4 Huxley RR, Shiell AW, Law CM. The role of size at birth and postnatal catch-up growth in determining systolic blood pressure: a systematic review of the literature. *J Hypertens* 2000;18:815-31.

5 Barker DJ, Osmond C, Golding J*, et al.* Growth in utero, blood pressure in childhood and adult life, and mortality from cardiovascular disease. *BMJ* 1989;298:564-7.

6 Ong KK, Ahmed ML, Emmett PM*, et al.* Association between postnatal catch-up growth and obesity in childhood: prospective cohort study. *BMJ* 2000;320:967-71.

7 Forsen T, Nissinen A, Tuomilehto J*, et al.* Growth in childhood and blood pressure in Finnish children. *J Hum Hypertens* 1998;12:397-402.

8 Jarvelin MR, Sovio U, King V*, et al.* Early life factors and blood pressure at age 31 years in the 1966 northern Finland birth cohort. *Hypertension* 2004;44:838-46.

9 Ben-Shlomo Y, McCarthy A, Hughes R*, et al.* Immediate postnatal growth is associated with blood pressure in young adulthood: the Barry Caerphilly Growth Study. *Hypertension* 2008;52:638-44.

10 Fabricius-Bjerre S, Jensen RB, Faerch K*, et al.* Impact of birth weight and early infant weight gain on insulin resistance and associated cardiovascular risk factors in adolescence. *PLoS One* 2011;6:e20595.

For peer review only - http://bmjopen.bmj.com/site/about/guidelines.xhtml

11 Bai J, Abdul-Rahman MF, Rifkin-Graboi A*, et al.* Population Differences in Brain Morphology and Microstructure among Chinese, Malay, and Indian Neonates. *PLoS One* 2012;7:e47816.

12 O'Tierney PF, Lewis RM, McWeeney SK*, et al.* Immune response gene profiles in the term placenta depend upon maternal muscle mass. *Reprod Sci* 2012;19:1041-56.

13 Inskip HM, Godfrey KM, Robinson SM*, et al.* Cohort profile: The Southampton Women's Survey. *Int J Epidemiol* 2006;35:42-8.

14 Whincup PH, Bruce NG, Cook DG*, et al.* The Dinamap 1846SX automated blood pressure recorder: comparison with the Hawksley random zero sphygmomanometer under field conditions. *J Epidemiol Community Health* 1992;46:164-9.

15 Cole TJ, Freeman JV, Preece MA. Body mass index reference curves for the UK, 1990. *Arch Dis Child* 1995;73:25-9.

16 Rolland-Cachera MF, Deheeger M, Maillot M*, et al.* Early adiposity rebound: causes and consequences for obesity in children and adults. *Int J Obes (Lond)* 2006;30 Suppl 4:S11-7.

17 Stettler N, Tershakovec AM, Zemel BS*, et al.* Early risk factors for increased adiposity: a cohort study of African American subjects followed from birth to young adulthood. *Am J Clin Nutr* 2000;72:378-83.

18 Qiu A, Fortier MV, Bai J*, et al.* Morphology and microstructure of subcortical structures at birth: A large-scale Asian neonatal neuroimaging study. *Neuroimage* 2012;65C:315-23.

19 Harvey NC, Mahon PA, Kim M*, et al.* Intrauterine growth and postnatal skeletal development: findings from the Southampton Women's Survey. *Paediatr Perinat Epidemiol* 2012;26:34-44.

20 Harvey N, Dhanwal D, Robinson S*, et al.* Does maternal long chain polyunsaturated fatty acid status in pregnancy influence the bone health of children? The Southampton Women's Survey. *Osteoporos Int* 2012;23:2359-67.

For peer review only

For peer review only - http://bmjopen.bmj.com/site/about/guidelines.xhtml

21 Jones A, Charakida M, Falaschetti E, *et al.* Adipose and height growth through childhood and blood pressure status in a large prospective cohort study. *Hypertension* 2012;59:919-25.

22 Chiolero A, Paradis G, Bovet P. Which period of growth is determinant for blood pressure? *Hypertension* 2012;60:e10; author reply e1.

23 Hindmarsh PC, Bryan S, Geary MP, *et al.* Effects of current size, postnatal growth, and birth size on blood pressure in early childhood. *Pediatrics* 2010;126:e1507-13.

24 Godfrey KM, Haugen G, Kiserud T, *et al.* Fetal liver blood flow distribution: role in human developmental strategy to prioritize fat deposition versus brain development. *PLoS One* 2012;7:e41759.

25 Singhal S, A. Early growth and later atherosclerosis. *World Rev Nutr Diet* 2013;106:162-7.

26 Wells JC, Chomtho S, Fewtrell MS. Programming of body composition by early growth and nutrition. *Proc Nutr Soc* 2007;66:423-34.

For peer review only

For peer review only - http://bmjopen.bmj.com/site/about/guidelines.xhtml

Figure 1

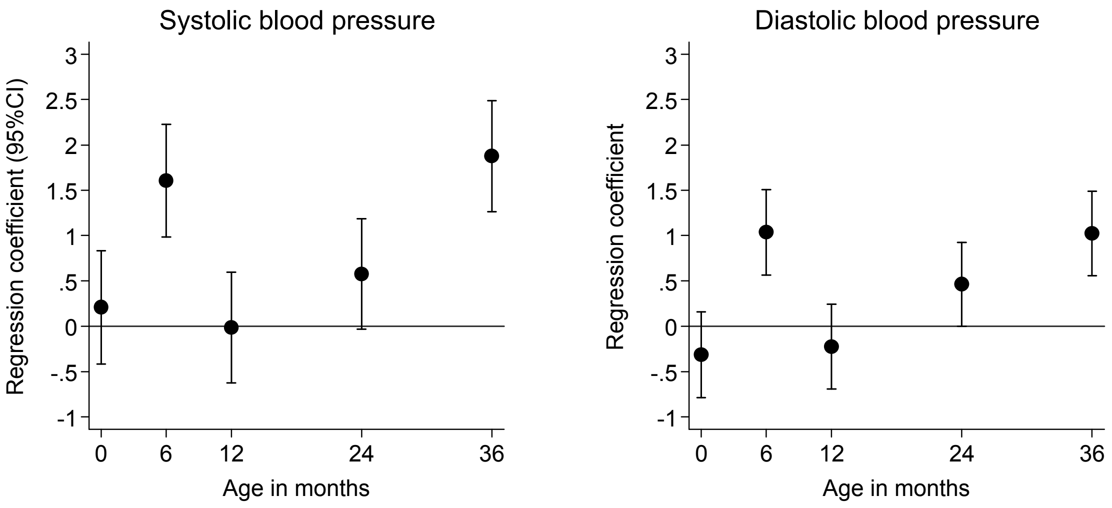

For peer review only

STROBE Statement—checklist of items that should be included in reports of observational studies

	Item No	Recommendation
**Title and abstract**	1	(*a*) Indicate the study's design with a commonly used term in the title or the abstract
		(*b*) Provide in the abstract an informative and balanced summary of what was done and what was found
**Introduction**		
Background/rationale	2	Explain the scientific background and rationale for the investigation being reported
Objectives	3	State specific objectives, including any prespecified hypotheses
**Methods**		
Study design	4	Present key elements of study design early in the paper
Setting	5	Describe the setting, locations, and relevant dates, including periods of recruitment, exposure, follow-up, and data collection
Participants	6	(*a*) *Cohort study*—Give the eligibility criteria, and the sources and methods of selection of participants. Describe methods of follow-up<br>*Case-control study*—Give the eligibility criteria, and the sources and methods of case ascertainment and control selection. Give the rationale for the choice of cases and controls<br>*Cross-sectional study*—Give the eligibility criteria, and the sources and methods of selection of participants
		(*b*) *Cohort study*—For matched studies, give matching criteria and number of exposed and unexposed<br>*Case-control study*—For matched studies, give matching criteria and the number of controls per case
Variables	7	Clearly define all outcomes, exposures, predictors, potential confounders, and effect modifiers. Give diagnostic criteria, if applicable
Data sources/ measurement	8*	For each variable of interest, give sources of data and details of methods of assessment (measurement). Describe comparability of assessment methods if there is more than one group
Bias	9	Describe any efforts to address potential sources of bias
Study size	10	Explain how the study size was arrived at
Quantitative variables	11	Explain how quantitative variables were handled in the analyses. If applicable, describe which groupings were chosen and why
Statistical methods	12	(*a*) Describe all statistical methods, including those used to control for confounding
		(*b*) Describe any methods used to examine subgroups and interactions
		(*c*) Explain how missing data were addressed
		(*d*) *Cohort study*—If applicable, explain how loss to follow-up was addressed<br>*Case-control study*—If applicable, explain how matching of cases and controls was addressed<br>*Cross-sectional study*—If applicable, describe analytical methods taking account of sampling strategy
		(*e*) Describe any sensitivity analyses

Continued on next page

For peer review only - http://bmjopen.bmj.com/site/about/guidelines.xhtml

**Results**		
Participants	13*	(a) Report numbers of individuals at each stage of study—eg numbers potentially eligible, examined for eligibility, confirmed eligible, included in the study, completing follow-up, and analysed
		(b) Give reasons for non-participation at each stage
		(c) Consider use of a flow diagram
Descriptive data	14*	(a) Give characteristics of study participants (eg demographic, clinical, social) and information on exposures and potential confounders
		(b) Indicate number of participants with missing data for each variable of interest
		(c) *Cohort study*—Summarise follow-up time (eg, average and total amount)
Outcome data	15*	*Cohort study*—Report numbers of outcome events or summary measures over time
		Case-control study—Report numbers in each exposure category, or summary measures of exposure
		Cross-sectional study—Report numbers of outcome events or summary measures
Main results	16	(*a*) Give unadjusted estimates and, if applicable, confounder-adjusted estimates and their precision (eg, 95% confidence interval). Make clear which confounders were adjusted for and why they were included
		(*b*) Report category boundaries when continuous variables were categorized
		(*c*) If relevant, consider translating estimates of relative risk into absolute risk for a meaningful time period
Other analyses	17	Report other analyses done—eg analyses of subgroups and interactions, and sensitivity analyses
**Discussion**		
Key results	18	Summarise key results with reference to study objectives
Limitations	19	Discuss limitations of the study, taking into account sources of potential bias or imprecision. Discuss both direction and magnitude of any potential bias
Interpretation	20	Give a cautious overall interpretation of results considering objectives, limitations, multiplicity of analyses, results from similar studies, and other relevant evidence
Generalisability	21	Discuss the generalisability (external validity) of the study results
**Other information**		
Funding	22	Give the source of funding and the role of the funders for the present study and, if applicable, for the original study on which the present article is based

*Give information separately for cases and controls in case-control studies and, if applicable, for exposed and unexposed groups in cohort and cross-sectional studies.

Note: An Explanation and Elaboration article discusses each checklist item and gives methodological background and published examples of transparent reporting. The STROBE checklist is best used in conjunction with this article (freely available on the Web sites of PLoS Medicine at http://www.plosmedicine.org/, Annals of Internal Medicine at http://www.annals.org/, and Epidemiology at http://www.epidem.com/). Information on the STROBE Initiative is available at www.strobe-statement.org.

For peer review only

BMJ Open

Association of early childhood abdominal circumference and weight gain with blood pressure at 36 months of age: secondary analysis of data from a prospective cohort study

Journal:	*BMJ Open*
Manuscript ID:	bmjopen-2014-005412.R1
Article Type:	Research
Date Submitted by the Author:	06-Jun-2014
Complete List of Authors:	Nowson, Caryl; Deakin University, Centre for Physical Activity and Nutrition Research<br>Crozier, S; University of Southampton & Southampton General Hospital, MRC Lifecourse Epidemiology Unit<br>Robinson, S; University of Southampton & Southampton General Hospital, MRC Lifecourse Epidemiology Unit<br>Godfrey, Keith; University of Southampton & Southampton General Hospital, MRC Lifecourse Epidemiology Unit; University of Southampton & University Hospital Southampton NHS Foundation Trust, NIHR Southampton Biomedical Research Centre<br>Lawrence, Wendy; University of Southampton & Southampton General Hospital, MRC Lifecourse Epidemiology Unit<br>Law, Catherine; University College London, UCL Institute of Child Health<br>Cooper, Cyrus; University of Southampton & Southampton General Hospital, MRC Lifecourse Epidemiology Unit<br>Inskip, Hazel; University of Southampton & Southampton General Hospital, MRC Lifecourse Epidemiology Unit
**Primary Subject Heading**:	Paediatrics
Secondary Subject Heading:	Epidemiology, Cardiovascular medicine, Nutrition and metabolism
Keywords:	Hypertension < CARDIOLOGY, Cardiac Epidemiology < CARDIOLOGY, EPIDEMIOLOGY

SCHOLARONE™ Manuscripts

Association of early childhood abdominal circumference and weight gain with blood pressure at 36 months of age: secondary analysis of data from a prospective cohort study

Caryl A Nowson[1], Sarah R Crozier[2], Siân M Robinson[2], Keith M Godfrey[2,3], Wendy T Lawrence[2], Catherine M Law[4], Cyrus Cooper[2] and Hazel M Inskip[2]

[1]Centre of Physical Activity and Nutrition Research, School of Exercise and Nutrition Sciences, Deakin University, Burwood, Victoria, Australia

[2]MRC Lifecourse Epidemiology Unit, University of Southampton & Southampton General Hospital, Southampton, UK

[3]NIHR Southampton Biomedical Research Centre, University of Southampton & University Hospital Southampton NHS Foundation Trust, Southampton, UK

[4]MRC Centre of Epidemiology for Child Health/Centre for Paediatric Epidemiology and Biostatistics, UCL Institute of Child Health, London, UK

Corresponding author:

Professor Caryl A. Nowson, Centre for Physical Activity and Nutrition Research, Deakin University, Locked Bag 200000, Geelong, VIC. 3220 caryl.nowson@deakin.edu.au , Tel: +61 3 5247 9245, Fax: +61 3 5227 8376

Key words: paediatrics, growth and development, blood pressure, body weight, abdominal circumference

Abbreviations: BMI: body mass index; CI: confidence interval; m: months, SWS: Southampton Women's Survey

Wordcount: 3,044.

For peer review only

For peer review only - http://bmjopen.bmj.com/site/about/guidelines.xhtml

ABSTRACT

Objectives: To assess if changes in measures of fat distribution and body size during early life are associated with blood pressure at 36 months of age.

Design: Analysis of data collected from a prospective cohort study.

Setting: Community-based investigation in Southampton, UK.

Participants: 761 children with valid blood pressure measurements, born to women participating in the Southampton Women's Survey.

Primary and secondary outcome measures: Anthropometric measurements were collected at 0, 6, 12, 24 and 36 months (mo) and conditional changes between the time-points calculated. Blood pressure was measured at 36 mo. Factors possibly influencing the blood pressure were assessed using linear regression. All independent variables of interest and confounding variables were included in stepwise multiple regression to identify the model that best predicted blood pressure at 36mo.

Results: Greater conditional gains in abdominal circumference (AC) between 0–6 and 24–36mo were associated with higher systolic and diastolic blood pressures at 36mo (P<0.001). Subscapular skinfold and height gains were weakly associated with higher blood pressures, while greater weight gains between 0–6, 12–24 and 24–36mo were more strongly associated, but the dominant influences were abdominal circumference gains, particularly from 0-6mo and 24-36mo. Thus one standard deviation score (SDS) increases in AC between 0-6mo and between 24–36mo were associated with 1.59 mmHg (95% CI: 0.97, 2.21) and 1.84 mmHg (1.24, 2.46) higher systolic blood pressures, respectively, and 1.04 mmHg (0.57, 1.51) and 1.02 mmHg (0.56, 1.48) higher diastolic pressures, respectively.

Conclusions: Conditional gains in abdominal circumference, particularly within six months of birth and in the year preceding measurement, were more positively associated with blood pressure at

For peer review only

36mo than gains in other anthropometric measures. Above-average abdominal circumference gains in early childhood may contribute to adult hypertension and increased cardiovascular disease risk.

Strengths and Limitations of this Study

- This is one of few studies that have investigated detailed anthropometric changes in relation to blood pressure in early age and examined conditional changes between different age-points.
- Key confounding risk factors were adjusted for in the models, including maternal education attainment and smoking during pregnancy.
- A large number of children from a cross-section of socioeconomic backgrounds were included in the study.
- We were not able to include all the children born in the course of the cohort study as blood pressure measurements were not available for all children, but the study sample was found to be similar to the larger group at 36 months of age.
- Abdominal girth at this young age may only represent a gross measure of central fat deposition and differences between individuals may represent genetically/prenatally-determined differences in physique.

For peer review only - http://bmjopen.bmj.com/site/about/guidelines.xhtml

INTRODUCTION

Low birth weight and rapid postnatal weight gain have been linked to increased risk of cardiovascular disease,[1] obesity, and the metabolic syndrome - including hypertension[2] and insulin resistance[3] - later in life. Accelerated weight gain, characterised by above-average velocities of skeletal and non-skeletal postnatal growth, has been associated with higher blood pressure in childhood.[4] Low birth weight predicts blood pressure in later life,[5] but it is not clear how much this association can be attributed to low birth weight independently of accelerated postnatal weight gain, as infants who are born small for gestational age tend to gain weight more rapidly during the early postnatal period.[6]

It is thought that there may be critical periods at specific time-points early in life when accelerated growth predisposes to hypertension later in life.[7-10] Furthermore, rapid increase in weight-for-length in the first 6 months has been associated with higher systolic blood pressure in 3-year-olds.[11] Few studies have assessed indicators of body fat distribution in infants and young children. Body fat distribution has been associated with risk factor scores for cardiovascular risk in young children[12] and postnatal rapid weight gain has been linked to deposition of fat centrally in children at 5 years.[6]

Therefore, insight into whether postnatal alterations in body composition influence blood pressure in early childhood is relevant to the development of preventative strategies to reduce the risk of cardiovascular disease in later life. Our aim was to assess how gains in adiposity, fat distribution and body size between birth, 6, 12, 24 and 36 months relate to the blood pressure of children at 36 months.

For peer review only - http://bmjopen.bmj.com/site/about/guidelines.xhtml

METHODS

Study sample: the Southampton Women's Survey (SWS)

The SWS is a large prospective cohort study which commenced in 1998.[13] A total of 12,583 non-pregnant women aged 20 to 34 years were recruited to the study. Detailed information on diet and socio-demographic factors was collected and children born to SWS women were assessed at birth and then followed up at home by trained research nurses. The SWS was approved by the Southampton and South West Hampshire Local Research Ethics Committee, and participants gave written informed consent. The research conformed to the principles embodied in the Declaration of Helsinki.

There were 1,981 singleton live births to women in the SWS by the end of 2003. After exclusion of infants with major congenital abnormalities (n=2) and neonatal deaths (n=6), 1,973 SWS infants remained for postnatal follow-up.

Maternal and child data

When each child was 24 months old, the occupations of its mother and her partner were recorded and the highest-ranking of these used to define the child's social class. The social class scale was: Professional (I), Management and technical (II), Skilled non-manual (IIIN), Skilled manual (IIIM), Partly skilled (IV), and Unskilled (V). For 10 children whose parental occupations were missing at this time, employment status recorded during early pregnancy was used. Educational attainment of the mother recorded before pregnancy was defined in six groups, from 'none' to 'degree or above'.

For peer review only - http://bmjopen.bmj.com/site/about/guidelines.xhtml

Body composition and blood pressure assessment

Anthropometric measurements were taken by trained researchers at birth, 6mo, 12mo, 24mo and 36mo. Apart from those at birth, all measurements were taken in the children's homes. Infant crown-heel length was measured with a neonatometer (CMS Ltd, London, United Kingdom). Child height was measured with a portable stadiometer (Leicester height measurer; CMS Ltd). Skinfold thicknesses were measured using Holtain skinfold callipers (Holtain Ltd) at specified sites, and abdominal circumference measured at the end of expiration using a blank tape measured against a fixed scale. Strict monitoring of the nurses' measurement techniques was performed by the senior research nurse and regular inter-observer variation studies were conducted.

Blood pressure was measured using a Critikon DINAMAP 1846 SX automated blood pressure device [14] with the child seated. Three measurements were recorded and the average of the last two used in the analysis. Due to limited equipment availability, blood pressure measurements were only available for approximately 47% of the SWS children.

Statistical analysis

Regression coefficients (β), with associated 95 % confidence intervals (CI), were used to assess the strength of association between body size indicators (body weight, length/height, abdominal circumference and subscapular skinfold thickness). Z-scores were calculated for body weight and length/height using the 1990 British growth references for time points 6mo, 12mo, 24mo and 36mo.[15] Z-scores for abdominal circumference and subscapular skinfolds were calculated internally using the SWS sample and were adjusted for gender, current age and gestational age. Conditional growth was derived from the residuals resulting from regression of the z-score for the measurement at a specific time point on the z-scores for measurements at all preceding ages. For example, the

For peer review only - http://bmjopen.bmj.com/site/about/guidelines.xhtml

dependent variable ‘conditional gain in body weight from 12mo to 24mo’ was derived as the residual of the regression of body weight z-score at 24mo on the z-scores for body weight at 12mo, 6mo and birth.

Factors reported to be associated with blood pressure were assessed using linear regression, including: age, social class, maternal education attainment, smoking in late pregnancy (an indicator of smoking throughout pregnancy) and crying of the child during blood pressure measurement. Factors that were univariately associated with blood pressure at age 36mo were retained for inclusion in regression models, namely crying, smoking and education.

Multiple regression analysis was performed by entering all independent variables of interest into the model, in addition to the confounding variables. A stepwise multiple regression analysis was used to identify the growth variables that were most strongly associated with blood pressure at 36mo. Statistical analyses were performed using SPSS PASW Statistics Release 18 (IBM SPSS, IBM Corp, New York), and Stata 12.0 (StataCorp, Texas, USA).

RESULTS

At 36mo of age, 1,640 children (83% of the 1,973 available for follow-up) were followed-up. Birth weights and 36mo blood pressure measurements were available for 773 infants. Seven children with missing height and weight data at age 36mo and five with systolic pressures more than 3 standard deviations from the mean were excluded, leaving 761 in the analysis. Children in this study were similar to the larger population sample of SWS children seen at 36mo (Table 1). Owing to a relative unavailability of blood pressure machines during later fieldwork, children included in the analyses were more likely to have been visited earlier in the study, and their mothers were slightly

For peer review only

For peer review only - http://bmjopen.bmj.com/site/about/guidelines.xhtml

younger. Additionally, compared with those not included, those children in the study were marginally older (by approximately 1 week), and lighter and shorter at birth, and their mothers were of lower social class, had lower educational qualifications and were more likely to have been smoking in late pregnancy. The full ranges of social classes and educational levels were represented in the analysis sample, although the 'Professional/Management & technical' social class accounted for around 40% of the population, and just over half the mothers had completed higher school/post-school qualifications.

For peer review only

For peer review only - http://bmjopen.bmj.com/site/about/guidelines.xhtml

Table 1. Maternal and infant characteristics of the Southampton Women's Survey study group

	With BP measurement (n=761)		Without BP measurement (n=879)	
**Mothers**	Mean	SD	Mean	SD
Maternal age at birth of the child***	29.7	3.7	30.6	3.8
Pre-pregnancy weight (kg)	67.3	13.9	68.3	13.9
Height (cm)	163.0	6.5	163.4	6.3
Maternal BMI (pre-pregnancy) (kg/m^2)	25.3	4.8	25.5	4.8
	Percent		Percent	
Smoking in pregnancy*	16.2		12.2	
Social class				
Professional/Management & technical (I/II)	39.2		43.0	
Skilled manual/non-manual (III)	48.4		46.5	
Partly skilled/unskilled (IV/V)	12.4		10.6	
Educational attainment*				
Compulsory education to age 16 years	44.9		39.0	
Post compulsory education	55.1		61.0	
**Infants**				
Gender - male, n (%)	397(52.2)		473 (53.8)	
	Mean	SD	Mean	SD
Age in years at 36mo visit ***	3.09	0.10	3.07	0.09
Weight (kg) at 36mo visit	15.0	1.9	15.1	1.8
Height (cm) at 36mo visit	95.7	3.7	96.0	3.6

For peer review only

For peer review only - http://bmjopen.bmj.com/site/about/guidelines.xhtml

Abdominal circumference (cm) at 36mo visit	51.2	3.2	51.3	3.1
Subscapular skinfold (mm) at 36mo visit	6.63	1.85	6.49	1.76
Systolic blood pressure (mmHg) at 36mo visit	93.8	8.3		
Diastolic blood pressure (mmHg) at 36mo visit	58.1	6.3		
Birth weight (kg)**	3.42	0.57	3.50	0.52
Birth length (cm)*	49.8	2.1	50.0	2.0

BP: blood pressure, SD: standard deviation, BMI: body mass index
*P<0.05, **P<0.01, ***P<0.001

For peer review only

The greatest relative and absolute increases in body weight, height/length, abdominal circumference and subscapular skinfold thickness occurred between birth and 6mo (Table 2), with the other age intervals (6-12mo, 12-24mo and 24-36mo) indicating smaller positive increments for body weight and height. Mean values of height and weight were comparable to the 50th percentile.[15] For subscapular skinfold, average changes between later ages from 6m were negative, as was the average change in abdominal circumference between 24 and 36mo. During this final age period, abdominal circumference increased in 39% of children but decreased in 60%.

Confounding variables

Age, gender and social class were not associated with blood pressure, but the 45 infants who cried during measurement had higher systolic (P=0.004) and diastolic pressures (P=0.001). Smoking in late pregnancy was associated with higher systolic (P=0.067) and diastolic pressures (p=0.005). Lower educational attainment (P=0.024) was associated with higher diastolic pressure.

For peer review only

For peer review only - http://bmjopen.bmj.com/site/about/guidelines.xhtml

Table 2. Body composition at 0, 6, 12, 24, and 36 months and incremental changes

				Absolute change		% change[1]	
	Mean	SD		Mean	SD	Mean	SD
N=712							
Birth weight (kg)	3.4	0.6	*Δ wt (kg): 0 - 36mo*	*11.6*	*1.8*	*349.7*	*92.7*
Weight (kg) 6mo	7.9	1.0	Δ wt (kg): 0 - 6mo	4.4	0.9	134.4	43.6
Weight (kg) 12mo	10.0	1.2	Δ wt (kg): 6 - 12mo	2.1	0.6	27.2	8.2
Weight (kg) 24mo	12.5	1.5	Δ wt (kg):12 - 24mo	2.6	0.9	26.0	8.5
Weight (kg) 36mo	15.0	1.9	Δ wt (kg): 24 - 36mo	2.5	0.9	20.1	7.0
N=666							
Birth length (cm)	49.8	2.1	*Δ ht (cm): 0 - 36mo*	*46*	*3.2*	*92.7*	*8.3*
Height (cm) 6mo	67.2	2.5	Δ ht (cm): 0 - 6mo	17.4	2.1	35.1	5.0
Height (cm) 12mo	75.6	2.7	Δ ht (cm): 6 - 12mo	8.4	1.8	12.6	2.8
Height (cm) 24mo	86.3	3.1	Δ ht (cm): 12 - 24mo	10.8	1.9	14.3	2.5
Height (cm) 36mo	95.8	3.6	Δ ht (cm): 24 - 36mo	9.5	1.6	11.0	1.9

For peer review only

For peer review only - http://bmjopen.bmj.com/site/about/guidelines.xhtml

N=682							
Abdominal circum.[2](cm) 0mo	31.5	2.2	*Δ circum. (cm): 0 - 36mo*	*19.8*	*3.1*	*63.3*	*12.3*
Abdominal circum. (cm) 6mo	47.4	3.2	Δ circum. (cm): 0 - 6mo	15.9	3.3	50.9	12.3
Abdominal circum.(cm) 12mo	49.6	3.2	Δ circum. (cm): 6 -12mo	2.2	2.6	4.7	5.7
Abdominal circum. (cm) 24mo	51.9	3.4	Δ circum. (cm): 12 - 24mo	2.3	2.9	4.8	6.0
Abdominal circum.(cm) 36mo	51.3	3.1	Δ circum. (cm): 24 - 36mo	-0.6	2.5	-1.0	4.7
N=645							
Subscapular skinfold (mm) 0mo	5.0	1.0	*Δ subscap[3].(mm): 0 - 36mo*	*1.7*	*1.9*	*37.2*	*42.4*
Subscapular skinfold (mm) 6mo	7.4	1.6	Δ subscap. (mm): 0 - 6mo	2.4	1.7	52.7	41.7
Subscapular skinfold (mm) 12mo	7.2	1.6	Δ subscap. (mm): 6- 12mo	-0.2	1.5	-1.8	19.3
Subscapular skinfold (mm) 24mo	6.6	1.6	Δ subscap. (mm): 12 -24mo	-0.6	1.4	-7.3	19.3
Subscapular skinfold (mm) 36mo	6.7	1.9	Δ subscap. (mm): 24 -36mo	0.1	1.4	3.6	20.1

SD: standard deviation

[1] calculated from previous time interval [2]abdominal circumference [3]subscapular skinfold thickness

For peer review only

Associations with blood pressure at 36 months

Initially, the four anthropometric measurements were considered separately (Table 3). Each model contained the measurement at birth and the conditional changes in the measure over the four age periods (0-6mo, 6-12mo, 12-24mo, and 24-36mo), along with the confounding factors that contributed significantly to the regression analysis; the slope represents the change in blood pressure (mmHg) per SD change in growth measurement. In all four models, the measurements at birth were not associated with blood pressure, independently of measurements of postnatal growth. The model for abdominal circumference explained more of the variance in blood pressure (8.8% and 7.7%, respectively, for systolic and diastolic blood pressure) (Figure 1) than the models for the other three measures, with the model for weight coming a close second (6.7% and 5.5% of the variance explained for systolic and diastolic blood pressure). The change in abdominal circumference closest to the blood pressure measurement, 24-36mo, was related most strongly to blood pressure (both systolic and diastolic), but change during the first six months of life was also significantly associated with both systolic and diastolic blood pressure. Weight change between birth and 6mo was related to blood pressure at 36mo, but weight change between 12 and 24mo also appeared to influence both systolic and diastolic blood pressure. Neither height nor subscapular skinfold thickness changes were related to blood pressure as strongly as abdominal circumference or weight changes, although for diastolic blood pressure there was a robust association with height change between 12 and 24mo. The effect sizes for the significant associations of body fat distribution and body weight were such that a 1 SDS increase in the measurement between the two ages under consideration was associated with an increase of around 1-2 mmHg in systolic blood pressure and approximately 1 mmHg in diastolic pressure

For peer review only

Table 3. Multiple regression models: associations between conditional gain (z-scores) in body composition measures and blood pressure[1,2]

	Systolic Pressure					Diastolic Pressure				
	Adj.R^2	β	95% CI		P- value	Adj.R^2	β	95% CI		P-value
**Model 1 – Weight[3] (N=684)**	0.067					.055				
Birth weight z-score		0.22	-0.45	0.89	0.5		0.12	-0.38	0.62	0.6
Weight: 0-6mo[4]		1.40	0.76	2.02	<0.001		0.81	0.34	1.27	0.001
Weight: 6-12mo[4]		0.51	-0.09	1.12	0.1		0.36	-0.09	0.82	0.1
Weight: 12-24mo[4]		1.20	0.59	1.81	<0.001		0.76	0.30	1.22	0.001
Weight: 24-36mo[4]		1.07	0.46	1.67	0.001		0.44	-0.01	0.89	0.06
**Model 2 – Length/height[3] (N=649)**	0.027					.038				
Birth length z-score		0.41	-0.36	1.18	0.3		0.08	-0.49	0.65	0.8
Height: 0-6mo[4]		0.91	0.27	1.54	0.005		0.42	-0.05	0.89	0.08
Height: 6-12mo[4]		0.66	0.02	1.30	0.04		0.27	-0.20	0.74	0.3
Height: 12-24mo[4]		0.74	0.09	1.38	0.03		0.72	0.25	1.20	0.003
Height: 24-36mo[4]		0.32	-0.33	0.96	0.3		0.15	-0.32	0.63	0.5

For peer review only

**Model 3 – Abdominal circumference[5] (N=664)**	.088					.077				
Birth abdominal circumference z-score		0.22	-0.40	0.84	0.5		-0.29	-0.76	0.18	0.2
Abdominal circumference[4]: 0-6mo		1.59	0.97	2.21	<0.001		1.04	0.57	1.51	<0.001
Abdominal circumference[4]: 6-12mo		-0.03	-0.64	0.59	0.9		-0.23	-0.70	0.23	0.3
Abdominal circumference[4]: 12-24mo		0.58	-0.03	1.19	0.06		0.46	-0.005	0.92	0.053
Abdominal circumference[4]: 24-36mo		1.84	1.24	2.46	<0.001		1.02	0.56	1.48	<0.001
**Model 4 – Subscapular skinfold thickness[5] (N=630)**	0.018					.035				
Birth subscapular skinfold		-0.17	-0.85	0.52	0.6		-0.25	-0.76	0.26	0.3
Subscapular skinfold[4]: 0-6mo		0.35	-0.33	1.02	0.3		0.09	-0.41	0.59	0.7
Subscapular skinfold[4]: 6 - 12mo		0.99	0.32	1.65	0.004		0.79	0.30	1.28	0.002
Subscapular skinfold[4]: 12 - 24mo		0.52	-0.14	1.17	0.1		0.02	-0.46	0.50	0.9
Subscapular skinfold[4]: 24 - 36mo		0.54	-0.12	1.19	0.1		0.25	-0.24	0.73	0.3

CI: confidence interval

[1]residuals derived from regression model with the specified z-score as the independent variable, [2]all analyses adjusted for crying, maternal education and maternal smoking in late pregnancy, [3]z-scores derived from percentile curve growth charts UK [15], [4]adjusted for measurements at all preceding time points, [5]z-scores derived internally from Southampton Women's Survey data.

For peer review only

The results of the final regression models are presented in Table 4. In the combined model, both abdominal circumference (0-6mo and 24-36 mo) and weight change (12-24mo) remained significantly associated with systolic blood pressure. The growth variables contributing to the final models were not highly correlated, with all correlations being less than 0.15. There was considerable variability in the gains of abdominal mass between 24 to 36mo, where 39% of children experienced an increase in abdominal circumference. Abdominal circumference change in the age period leading up to the blood pressure measurement was the key influence on blood pressure, with a 1 SDS change in the circumference being associated with a 1.66mmHg increase in systolic blood pressure. The final model for diastolic blood pressure was similar, with key associations being abdominal circumference change in the earliest and latest age periods (0-6mo and 24-36mo) with a 1 SD increase in abdominal circumference change associated with approximately 1 mmHg higher diastolic blood pressure. After the abdominal circumference changes were included in the model, changes in weight no longer appeared to influence blood pressure, but height change between 12 and 24mo was retained in the model. The addition of the variables 'ever breast-fed' or 'duration of breast feeding' to the final models did not affect the relationships between blood pressure and body composition measures.

For peer review only

For peer review only - http://bmjopen.bmj.com/site/about/guidelines.xhtml

Table 4. Multivariate regression model of best fit: conditional gain (z-scores) in body size and fat distribution associations with blood pressure at 36 months[2]

**Systolic Pressure**					
N=650	Adj.R^2	β	95% CI		P-value
	.094				
Abdominal circumference[3,4]: 0-6mo		1.52	0.07	1.71	0.04
Abdominal circumference[3,4]: 24-36mo		1.66	0.94	2.27	<0.0001
Weight: 12-24mo[3,5]		0.79	0.16	1.39	0.01
**Diastolic Pressure**					
N=625	Adj.R^2	β	95% CI		P-value
	.076				
Abdominal circumference[3,4]: 0-6mo		0.95	0.48	1.42	<0.001
Abdominal circumference[3,4]: 24-36mo		0.94	0.46	1.43	<0.001
Height: 12-24mo[3,5]		0.60	0.12	1.08	.015

CI: confidence interval

[1]residuals derived from regression model with the specified z-score as the independent variable, [2]all analyses adjusted for crying, maternal education and maternal smoking in late pregnancy, [3]adjusted for all the preceding time intervals (0-6mo, 6-12mo, 12-24mo, 24-36mo), [4]Z-scores based on sample population data from Southampton Women's Survey, [5]z-scores derived from percentile curve growth charts UK [15]

For peer review only - http://bmjopen.bmj.com/site/about/guidelines.xhtml

DISCUSSION

Main findings

Our data indicate that conditional gains in abdominal circumference between birth and 6 months, as well as in the 12 months prior to blood pressure measurement, were associated with both systolic and diastolic blood pressure at 36 months of age. Changes in weight were also associated with blood pressure, particularly systolic pressure, but to a lesser extent. In the multivariate regression model, a one-SD gain in abdominal circumference z-score between 24 and 36mo was associated with a 1.66 mmHg increase in systolic blood and a one-SD increase between birth and 6mo with 1.56mmHg.

There was considerable variability in the gains of abdominal mass at the time interval most predictive of blood pressure (24 to 36mo), with 39% of children experiencing an increase, suggesting the impact of environmental factors. It is possible that dietary intake and/or physical activity levels during this early period could alter abdominal circumference gain early in life, or it may be related to differences in growth trajectories,[16] which predict later obesity. Given that abdominal circumference in the majority of children decreases between the ages of 24 and 36mo, increases seen in this time period might be an early marker for problems in later life, particularly if the effect of abdominal circumference change on blood pressure amplifies with age.

It is of note that subscapular skinfold thickness change, an indicator of subcutaneous fat deposition, had much weaker associations with blood pressure than abdominal circumference, which is an indicator of abdominal or visceral fat deposition. The finding that abdominal circumference gain between 0-6mo was more strongly associated with blood pressure at age 36mo than gains during 6-12mo and 12-24mo could reflect the proposed critical period for childhood adiposity in the first two months of life,[17] or could reflect gains in adiposity associated with an adverse intrauterine environment in late gestation.

For peer review only - http://bmjopen.bmj.com/site/about/guidelines.xhtml

Strengths and weaknesses

As summarised earlier, a strength of this study is the large sample size drawn from the general population. A post-hoc power calculation (n=650, with SD of 8mmHg for systolic pressure) indicated there was 80% power to detect a regression coefficient of 0.88mmHg per 1SD of an independent variable at 5% level of significance. Our study was thus sufficiently large to detect effect sizes of clinical relevance. In addition, we have assessed conditional growth, accounting for measurements at previous ages (thus ensuring independence of growth summaries between age periods), and have adjusted for relevant confounding factors.

Few studies have collected such detailed anthropometric measurements with the methodological rigor to provide indicators of fat distribution in the early postnatal period, encompassing the transition from milk-only to a mixed diet at the age of 36 months.

It is clear from the data that the strongest association with blood pressure from all the anthropometric measurements is the gain in abdominal circumference; however, we acknowledge that when building a multivariate regression model of best fit, there is an element of chance as to which variables are included, even when they are not highly correlated.

A limitation of the study is the inability to measure blood pressure in all children in the cohort, although there do not appear to be noteworthy differences between those who were measured and the remainder.

For peer review only - http://bmjopen.bmj.com/site/about/guidelines.xhtml

Comparison with other studies

Central adiposity and a large waist circumference have been associated with higher blood pressure in adults, but there are few reports for children. In one study, children with accelerated weight gain in the first two years of life were fatter and had more central fat distribution at five years of age than other children.[6] However, the relationship of central fat deposition specifically to blood pressure, which is only one component of the metabolic syndrome, is not clear in children. Rapid weight gain during infancy (0–6mo) but not during early childhood (3–6 years) was associated with a higher metabolic risk score at age 17 years, but the association between waist circumference and blood pressure was not statistically significant in that study.[18] Waist-for-height ratio in school children has not been found to confer additional discriminative power to BMI in some studies,[19, 20] but recently weight and weight-for-height changes through infancy and childhood have been associated with blood pressure at age 10 years[21] and at age 9.1 and 15.5 years.[22] These studies did not consider the periods 0-6mo and 6-12mo separately, nor examine change in abdominal circumference. However, similar to both reports, we found that weight changes at ages closest to those at which the blood pressure measurement was made were most strongly associated.

There is considerable debate as to whether there are specific age-periods early in life during which weight gain and/or fat deposition are more influential in predicting cardiovascular risk. Our findings are similar to a report that weight gain between 24 and 36mo was positively associated with systolic blood pressure.[23] The mean blood pressure values in our study of 94/58 mmHg are similar to those reported (92/58 mmHg),[23] as are the correlations between systolic blood pressure and current weight and height at 36mo. Similarly, we also did not find an inverse association between birth weight and blood pressure. However, in contrast we found that conditional gains in body weight between all age periods were positively associated with systolic blood pressure and a similar trend for diastolic pressure, except for the period between 6 to 12mo. The

For peer review only - http://bmjopen.bmj.com/site/about/guidelines.xhtml

differences in results may be related to our adjustment for all previous time intervals when we determined conditional growth.

Our data support a developmental contribution to the origin of elevated blood pressure in childhood, and therefore potentially in later adulthood. Indeed, we have previously demonstrated that higher fetal liver blood flow is strongly correlated with greater fat mass at birth and at 4 years, indicating that influences on body composition operate very early in life.[24] Our data show that changes in abdominal circumference during the first six months and in the third year of life are associated with systolic and diastolic blood pressure. This suggests that the tracking of blood pressure, evident by later childhood, might be a consequence of under-supply of conditionally essential nutrients associated with elevated fetal liver blood flow and prioritisation of fat deposition in the infant.

It is not clear whether postnatal interventions would be effective in limiting growth in abdominal circumference, and any interventions to limit abdominal circumference gains would likely also reduce the rate of growth. However, there is accumulating evidence that accelerated early growth has long-term adverse physiological effects in later life, increasing cardiovascular risk factors, including high blood pressure.[25] Promotion of lifestyle practices, such as exclusive breast feeding up to 6 months, later introduction of solid foods and maximising opportunities for safe physical activity, could contribute to a reduction in accelerated growth early in life and be one pathway that results in a reduction in cardiovascular disease later in life.

Conclusion

We have demonstrated that although conditional gains in body weight and height during specific age periods are associated with systolic and diastolic blood pressure, as previously recognised, conditional gains in abdominal circumference (associated with central fat deposition) have the strongest association with blood pressure at 36 months of age. This is especially true for the first 6 months of life and between 24 and 36

For peer review only - http://bmjopen.bmj.com/site/about/guidelines.xhtml

months (i.e., the year prior to blood pressure measurement). Therefore, central deposition of fat in early childhood, indicated by abdominal circumference, may contribute to an increased risk of developing hypertension later in life. Follow-up blood pressure measurements for the study group may enable confirmation of this at a later date.

For peer review only

For peer review only - http://bmjopen.bmj.com/site/about/guidelines.xhtml

Figure 1

Association between change in abdominal circumference (residuals derived from regressing the z-score at each specific age on the z-score at all preceding ages, adjusted for crying and maternal educational attainment and smoking during pregnancy) as the independent variable and blood pressure at 36 months (mmHg). Z-scores are based on data from the Southampton Women's Survey, adjusted for gender, current age and gestational age.

For peer review only

For peer review only - http://bmjopen.bmj.com/site/about/guidelines.xhtml

Acknowledgements

The Southampton Women's Survey Study Group includes Patsy Coakley, Vanessa Cox, Julia Hammond, Tina Horsfall and the Research Nurses who collected and processed the data for the Survey. The authors are very grateful to the families who participated in the research.

Competing Interests

None

Funding

This work was supported by grants from the Medical Research Council, British Heart Foundation, Arthritis Research UK, National Institute for Health Research (NIHR) Southampton Biomedical Research Centre, University of Southampton and University Hospital Southampton NHS Foundation Trust, and NIHR Musculoskeletal Biomedical Research Unit, University of Oxford. The work was also financially supported in part by the Commission of the European Community, specifically the RTD Programme "Quality of Life and Management of Living Resources", within the 7th Framework Programme, research grant no. FP7/2007-13 (Early Nutrition Project). This manuscript does not necessarily reflect the views of the funders and in no way anticipates the future policy in this area. KMG is supported by the National Institute for Health Research through the NIHR Southampton Biomedical Research Centre.

Author contributions

Caryl Nowson conceptualised the study, performed the analysis, contributed to the interpretation and drafted the initial manuscript. Sarah Crozier assisted with the analysis and contributed to the interpretation. Siân Robinson contributed to interpretation. Keith Godfrey, Wendy Lawrence, Catherine Law and Cyrus Cooper

For peer review only - http://bmjopen.bmj.com/site/about/guidelines.xhtml

reviewed and revised the manuscript. Hazel Inskip conceptualised the study and designed the analysis plan, performed analysis, contributed to the interpretation and revised the manuscript. All authors approved the final manuscript as submitted.

Data sharing

No additional data available.

For peer review only

For peer review only - http://bmjopen.bmj.com/site/about/guidelines.xhtml

REFERENCES

1 Ioannou C, Javaid MK, Mahon P*, et al.* The effect of maternal vitamin D concentration on fetal bone. *J Clin Endocrinol Metab* 2012;97:E2070-7.

2 Law CM, Shiell AW, Newsome CA*, et al.* Fetal, infant, and childhood growth and adult blood pressure: a longitudinal study from birth to 22 years of age. *Circulation* 2002;105:1088-92.

3 Noakes PS, Vlachava M, Kremmyda LS*, et al.* Increased intake of oily fish in pregnancy: effects on neonatal immune responses and on clinical outcomes in infants at 6 mo. *Am J Clin Nutr* 2012;95:395-404.

4 Huxley RR, Shiell AW, Law CM. The role of size at birth and postnatal catch-up growth in determining systolic blood pressure: a systematic review of the literature. *J Hypertens* 2000;18:815-31.

5 Barker DJ, Osmond C, Golding J*, et al.* Growth in utero, blood pressure in childhood and adult life, and mortality from cardiovascular disease. *BMJ* 1989;298:564-7.

6 Ong KK, Ahmed ML, Emmett PM*, et al.* Association between postnatal catch-up growth and obesity in childhood: prospective cohort study. *BMJ* 2000;320:967-71.

7 Forsen T, Nissinen A, Tuomilehto J*, et al.* Growth in childhood and blood pressure in Finnish children. *J Hum Hypertens* 1998;12:397-402.

8 Jarvelin MR, Sovio U, King V*, et al.* Early life factors and blood pressure at age 31 years in the 1966 northern Finland birth cohort. *Hypertension* 2004;44:838-46.

9 Ben-Shlomo Y, McCarthy A, Hughes R*, et al.* Immediate postnatal growth is associated with blood pressure in young adulthood: the Barry Caerphilly Growth Study. *Hypertension* 2008;52:638-44.

10 Fabricius-Bjerre S, Jensen RB, Faerch K*, et al.* Impact of birth weight and early infant weight gain on insulin resistance and associated cardiovascular risk factors in adolescence. *PLoS One* 2011;6:e20595.

For peer review only - http://bmjopen.bmj.com/site/about/guidelines.xhtml

11 Bai J, Abdul-Rahman MF, Rifkin-Graboi A, *et al.* Population Differences in Brain Morphology and Microstructure among Chinese, Malay, and Indian Neonates. *PLoS One* 2012;7:e47816.

12 O'Tierney PF, Lewis RM, McWeeney SK, *et al.* Immune response gene profiles in the term placenta depend upon maternal muscle mass. *Reprod Sci* 2012;19:1041-56.

13 Inskip HM, Godfrey KM, Robinson SM, *et al.* Cohort profile: The Southampton Women's Survey. *Int J Epidemiol* 2006;35:42-8.

14 Whincup PH, Bruce NG, Cook DG, *et al.* The Dinamap 1846SX automated blood pressure recorder: comparison with the Hawksley random zero sphygmomanometer under field conditions. *J Epidemiol Community Health* 1992;46:164-9.

15 Cole TJ, Freeman JV, Preece MA. Body mass index reference curves for the UK, 1990. *Arch Dis Child* 1995;73:25-9.

16 Rolland-Cachera MF, Deheeger M, Maillot M, *et al.* Early adiposity rebound: causes and consequences for obesity in children and adults. *Int J Obes (Lond)* 2006;30 Suppl 4:S11-7.

17 Stettler N, Tershakovec AM, Zemel BS, *et al.* Early risk factors for increased adiposity: a cohort study of African American subjects followed from birth to young adulthood. *Am J Clin Nutr* 2000;72:378-83.

18 Qiu A, Fortier MV, Bai J, *et al.* Morphology and microstructure of subcortical structures at birth: A large-scale Asian neonatal neuroimaging study. *Neuroimage* 2012;65C:315-23.

19 Harvey NC, Mahon PA, Kim M, *et al.* Intrauterine growth and postnatal skeletal development: findings from the Southampton Women's Survey. *Paediatr Perinat Epidemiol* 2012;26:34-44.

20 Harvey N, Dhanwal D, Robinson S, *et al.* Does maternal long chain polyunsaturated fatty acid status in pregnancy influence the bone health of children? The Southampton Women's Survey. *Osteoporos Int* 2012;23:2359-67.

For peer review only

21 Jones A, Charakida M, Falaschetti E, *et al.* Adipose and height growth through childhood and blood pressure status in a large prospective cohort study. *Hypertension* 2012;59:919-25.

22 Chiolero A, Paradis G, Bovet P. Which period of growth is determinant for blood pressure? *Hypertension* 2012;60:e10; author reply e1.

23 Hindmarsh PC, Bryan S, Geary MP, *et al.* Effects of current size, postnatal growth, and birth size on blood pressure in early childhood. *Pediatrics* 2010;126:e1507-13.

24 Godfrey KM, Haugen G, Kiserud T, *et al.* Fetal liver blood flow distribution: role in human developmental strategy to prioritize fat deposition versus brain development. *PLoS One* 2012;7:e41759.

25 Singhal S, A. Early growth and later atherosclerosis. *World Rev Nutr Diet* 2013;106:162-7.

26 Wells JC, Chomtho S, Fewtrell MS. Programming of body composition by early growth and nutrition. *Proc Nutr Soc* 2007;66:423-34.

For peer review only

For peer review only - http://bmjopen.bmj.com/site/about/guidelines.xhtml

~~Influence~~Association of early childhood abdominal circumference and weight gain ~~on~~with blood pressure at 36 months of age: secondary analysis of data from a prospective cohort study

Formatted: Top: 0.79"

Caryl A Nowson[1], Sarah R Crozier[2], Siân M Robinson[2], Keith M Godfrey[2,3], Wendy T Lawrence[2], Catherine M Law[4], Cyrus Cooper[2] and Hazel M Inskip[2]

[1]Centre of Physical Activity and Nutrition Research, School of Exercise and Nutrition Sciences, Deakin University, Burwood, Victoria, Australia

[2]MRC Lifecourse Epidemiology Unit, University of Southampton & Southampton General Hospital, Southampton, UK

[3]NIHR Southampton Biomedical Research Centre, University of Southampton & University Hospital Southampton NHS Foundation Trust, Southampton, UK

[4]MRC Centre of Epidemiology for Child Health/Centre for Paediatric Epidemiology and Biostatistics, UCL Institute of Child Health, London, UK

Corresponding author:

Professor Caryl A. Nowson, Centre for Physical Activity and Nutrition Research, Deakin University, Locked Bag 200000, Geelong, VIC. 3220 caryl.nowson@deakin.edu.au , Tel: +61 3 5247 9245, Fax: +61 3 5227 8376

Key words: paediatrics, growth and development, blood pressure, body weight, abdominal circumference

Abbreviations: BMI: body mass index; CI: confidence interval; m: months, SWS: Southampton Women's Survey

Wordcount: ~~2,956~~3,044.

For peer review only

For peer review only - http://bmjopen.bmj.com/site/about/guidelines.xhtml

ABSTRACT

Objectives: To assess if changes in measures of fat distribution and body size during early life are associated with blood pressure at 36 months of age.

Design: Analysis of data collected from a prospective cohort study.

Setting: Community-based investigation in Southampton, UK.

Participants: 761 children with valid blood pressure measurements, born to women participating in the Southampton Women's Survey.

Primary and secondary outcome measures: Anthropometric measurements were collected at 0, 6, 12, 24 and 36 months (mo) and conditional changes between the time-points calculated. Blood pressure was measured at 36 mo. Factors possibly influencing the blood pressure were assessed using linear regression. All independent variables of interest and confounding variables were included in stepwise multiple regression to identify the model that best predicted blood pressure at 36mo.

Results: Greater conditional gains in abdominal circumference (AC) between 0–6 and 24–36mo were associated with higher systolic and diastolic blood pressures at 36mo (P<0.001). Subscapular skinfold and height gains were weakly associated with higher blood pressures, while greater weight gains between 0–6, 12–24 and 24–36mo were more strongly associated, but the dominant influences were abdominal circumference gains~~. Systolic blood pressure increased by 0.87 mmHg (95% CI: 0.02,1.71) per~~, particularly from 0-6mo and 24-36mo. Thus one standard deviation score (SDS) ~~increase~~increases in ~~abdominal circumference~~AC between 0-6mo~~,~~ and ~~1.64 mmHg (1.02, 2.27)~~ between 24–36mo~~;~~ were associated with 1.59 mmHg (95% CI: 0.97, 2.21) and 1.84 mmHg (1.24, 2.46) higher systolic blood pressures, respectively, and 1.04 mmHg (0.57, 1.51) and 1.02 mmHg (0.56, 1.48) higher diastolic ~~pressure increased by 0.98 mmHg (0.51, 1.44) per SDS increase between 0–6mo, and 0.90 mmHg (0.43, 1.38) between 24–36mo.~~pressures, respectively.

For peer review only

Conclusions: Conditional gains in abdominal circumference, particularly within six months of birth and in the year preceding measurement, were more positively associated with blood pressure at 36mo than gains in other anthropometric measures. Above-average abdominal circumference gains in early childhood may contribute to adult hypertension and increased cardiovascular disease risk.

Strengths and Limitations of this Study

- This is one of few studies that have investigated detailed anthropometric changes in relation to blood pressure in early age and examined conditional changes between different age-points.
- Key confounding risk factors were adjusted for in the models, including maternal education attainment and smoking during pregnancy.
- A large number of children from a cross-section of socioeconomic backgrounds were included in the study.
- We were not able to include all the children born in the course of the cohort study as blood pressure measurements were not available for all children, but the study sample was found to be similar to the larger group at 36 months of age.
- Abdominal girth at this young age may only represent a gross measure of central fat deposition and differences between individuals may represent genetically/prenatally-determined differences in physique.

For peer review only - http://bmjopen.bmj.com/site/about/guidelines.xhtml

INTRODUCTION

Low birth weight and rapid postnatal weight gain have been linked to increased risk of cardiovascular disease,[1] obesity, and the metabolic syndrome - including hypertension[2] and insulin resistance[3] - later in life. Accelerated weight gain, characterised by above-average velocities of skeletal and non-skeletal postnatal growth, has been associated with higher blood pressure in childhood.[4] Low birth weight predicts blood pressure in later life,[5] but it is not clear how much this association can be attributed to low birth weight independently of accelerated postnatal weight gain, as infants who are born small for gestational age tend to gain weight more rapidly during the early postnatal period.[6]

It is thought that there may be critical periods at specific time-points early in life when accelerated growth predisposes to hypertension later in life.[7-10] Furthermore, rapid increase in weight-for-length in the first 6 months has been associated with higher systolic blood pressure in 3-year-olds.[11] Few studies have assessed indicators of body fat distribution in infants and young children. Body fat distribution has been associated with risk factor scores for cardiovascular risk in young children[12] and postnatal rapid weight gain has been linked to deposition of fat centrally in children at 5 years.[6]

Therefore, insight into whether postnatal alterations in body composition influence blood pressure in early childhood is relevant to the development of preventative strategies to reduce the risk of cardiovascular disease in later life. Our aim was to assess how gains in adiposity, fat distribution and body size between birth, 6, 12, 24 and 36 months relate to the blood pressure of children at 36 months.

For peer review only - http://bmjopen.bmj.com/site/about/guidelines.xhtml

METHODS

Study sample: the Southampton Women's Survey (SWS)

The SWS is a large prospective cohort study which commenced in 1998.[13] A total of 12,583 non-pregnant women aged 20 to 34 years were recruited to the study. Detailed information on diet and socio-demographic factors was collected and children born to SWS women were assessed at birth and then followed up at home by trained research nurses. The SWS was approved by the Southampton and South West Hampshire Local Research Ethics Committee, and participants gave written informed consent. The research conformed to the principles embodied in the Declaration of Helsinki.

There were 1,981 singleton live births to women in the SWS by the end of 2003. After exclusion of infants with major congenital abnormalities (n=2) and neonatal deaths (n=6), 1,973 SWS infants remained for postnatal follow-up.

Maternal and child data

When each child was 24 months old, the occupations of its mother and her partner were recorded and the highest-ranking of these used to define the child's social class. The social class scale was: Professional (I), Management and technical (II), Skilled non-manual (IIIN), Skilled manual (IIIM), Partly skilled (IV), and Unskilled (V). For 10 children whose parental occupations were missing at this time, employment status recorded during early pregnancy was used. Educational attainment of the mother recorded before pregnancy was defined in six groups, from 'none' to 'degree or above'.

For peer review only - http://bmjopen.bmj.com/site/about/guidelines.xhtml

Body composition and blood pressure assessment

Anthropometric measurements were taken by trained researchers at birth, 6mo, 12mo, 24mo and 36mo. Apart from those at birth, all measurements were taken in the children's homes. Infant crown-heel length was measured with a neonatometer (CMS Ltd, London, United Kingdom). Child height was measured with a portable stadiometer (Leicester height measurer; CMS Ltd). Skinfold thicknesses were measured using Holtain skinfold callipers (Holtain Ltd) at specified sites, and abdominal circumference measured at the end of expiration using a blank tape measured against a fixed scale. Strict monitoring of the nurses' measurement techniques was performed by the senior research nurse and regular inter-observer variation studies were conducted.

Blood pressure was measured using a Critikon DINAMAP 1846 SX automated blood pressure device [14] with the child seated. Three measurements were recorded and the average of the last two used in the analysis. Due to limited equipment availability, blood pressure measurements were only available for approximately 47% of the SWS children.

Statistical analysis

Regression coefficients (β), with associated 95 % confidence intervals (CI), were used to assess the strength of association between body size indicators (body weight, length/height, abdominal circumference and subscapular skinfold thickness). Z-scores were calculated for body weight and length/height using the 1990 British growth references for time points 6mo, 12mo, 24mo and 36mo.[15] Z-scores for abdominal circumference and subscapular skinfolds were calculated internally using the SWS sample and were adjusted for gender, current age and gestational age. Conditional growth was derived from the residuals resulting from regression of the z-score for the measurement at a specific time point on the z-scores for measurements at all preceding ages. For example, the

For peer review only

For peer review only - http://bmjopen.bmj.com/site/about/guidelines.xhtml

dependent variable 'conditional gain in body weight from 12mo to 24mo' was derived as the residual of the regression of body weight z-score at 24mo on the z-scores for body weight at 12mo, 6mo and birth.

Factors reported to be associated with blood pressure were assessed using linear regression, including;,: age, social class, maternal education attainment, smoking in late pregnancy (an indicator of smoking throughout pregnancy) and crying of the child during blood pressure measurement. Factors that were univariately associated with blood pressure at age 36mo were retained for inclusion in regression models that explored the relationship between blood pressure and body composition or fat distribution, but were subsequently dropped if they did not remain significant at the 5% level in the final models. , namely crying, smoking and education.

Multiple regression analysis was performed by entering all independent variables of interest into the model, in addition to the confounding variables. A stepwise multiple regression analysis was used to identify the model that best predictedgrowth variables that were most strongly associated with blood pressure at 36mo. Statistical analyses were performed using SPSS PASW Statistics Release 18 (IBM SPSS, IBM Corp, New York), and Stata 12.0 (StataCorp, Texas, USA).

RESULTS

At 36mo of age, 1,640 children (83% of the cohort1,973 available for follow-up) were followed-up. Birth weights and 36mo blood pressure measurements were then available for 773 infants. Seven children with missing height and weight data at age 36mo and five with systolic pressures more than 3 standard deviations from the mean were excluded, leaving 761 in the analysis. Children in this study were similar to the larger population sample of SWS children seen at

For peer review only - http://bmjopen.bmj.com/site/about/guidelines.xhtml

36mo (Table 1). Owing to a relative unavailability of blood pressure machines during later fieldwork, children included in the analyses were more likely to have been ~~studied earlier in the study.~~ visited earlier in the study, and their mothers were slightly younger. Additionally, compared with those not included, those children in the study were marginally older (by approximately 1 week), and lighter and shorter at birth, and their mothers were of lower social class, had lower educational qualifications and were more likely to have been smoking in late pregnancy. The full ranges of social classes and educational levels were represented in the analysis sample, although the 'Professional/Management & technical' social class accounted for around 40% of the population, and just over half the mothers had completed higher school/post-school qualifications.

For peer review only - http://bmjopen.bmj.com/site/about/guidelines.xhtml

Table 1. Maternal and infant characteristics of the Southampton Women's Survey study group

	With BP measurement (n=761)		Without BP measurement (n=879)	
**Mothers**	Mean	SD	Mean	SD
Maternal age at birth of the child***	29.7	3.7	30.6	3.8
WeightPre-pregnancy weight (kg)[1]	67.3	13.9	68.3	13.9
Height (mcm)	163.0	6.5	163.4	6.3
Maternal BMI (pre-pregnancy) (kg/m^2)[1]	25.3	4.8	25.5	4.8
	Percent		Percent	
Smoking in pregnancy*	16.02		12.02	
Social class				
Professional/Management & technical (I/II)	4039.2		4043.0	
Skilled manual/non-manual (III)	4348.4		45.946.5	
Partly skilled/unskilled (IV/V)	1612.4		14.110.6	
Educational attainment*				
Compulsory education to age 16 years	44.9		38.939.0	
Post compulsory education	55.1		65.161.0	
**Infants**				
Gender - male, n (%)	397(52.2)		473 (5453.8)	
	Mean	SD	Mean	SD
Age in years at 36mo visit***	3.109	0.110	3.107	0.109
Weight (kg) at 36mo visit	15.0	1.9	15.1	1.8
Height (cm) at 36mo visit	95.7	3.7	96.0	3.6

Formatted: Superscript

Formatted: Superscript

For peer review only

For peer review only - http://bmjopen.bmj.com/site/about/guidelines.xhtml

Abdominal circumference (cm) at 36mo visit	51.2	3.2	51.3	3.1
Subscapular skinfold (mm) at 36mo visit	6.663	1.885	6.549	1.876
Systolic blood pressure (mmHg) at 36mo visit	93.8	8.3		
Diastolic blood pressure (mmHg) at 36mo visit	58.1	6.3		
Birth weight (kg))**	3.442	0.657	3.550	0.552
Birth length (cm))*	49.8	2.1	50.0	2.0

BP: blood pressure, SD: standard deviation, BMI: body mass index

†based on reported weight 3 months prior to pregnancy

*P<0.05, **P<0.01, ***P<0.001

For peer review only - http://bmjopen.bmj.com/site/about/guidelines.xhtml

The greatest relative and absolute increases in body weight, height/length, abdominal circumference and subscapular skinfold thickness occurred between birth and 6mo (Table 2), with the other age intervals (6-12mo, 12-24mo and 24-36mo) indicating smaller positive increments for body weight and height. Mean values of height and weight were comparable to the -50th percentile.[15] For subscapular skinfold, average changes between later ages from 6m were negative, as was the average change in abdominal circumference between 24 and 36mo. During this final age period, abdominal circumference increased in 39% of children but decreased in 60%.

Confounding variables

Age, gender and social class were not associated with blood pressure, but the 45 infants who cried during measurement had higher systolic (P=0.004) and diastolic pressures (P=0.001). Smoking in late pregnancy was associated with higher systolic (P=0.067) and diastolic pressures (p=0.005). Lower educational attainment (P=0.024) was associated with higher diastolic pressure. ~~However, in the final models, smoking in late pregnancy was not significantly related to either systolic or diastolic pressure, so was dropped.~~

For peer review only - http://bmjopen.bmj.com/site/about/guidelines.xhtml

Table 2. Body composition at 0, 6, 12, 24, and 36 months and incremental changes

				Absolute change		% change[1]	
	Mean	SD		Mean	SD	Mean	SD
N=712							
Birth weight (kg)	3.4	0.6	*Δ wt (kg): 0 - 36mo*	*11.6*	*1.8*	*349.7*	*92.7*
Weight (kg) 6mo	7.9	1.0	Δ wt (kg): 0 - 6mo	4.4	0.9	134.4	43.6
Weight (kg) 12mo	10.0	1.2	Δ wt (kg): 6 - 12mo	2.1	0.6	27.2	8.2
Weight (kg) 24mo	12.5	1.5	Δ wt (kg):12 - 24mo	2.6	0.9	26.0	8.5
Weight (kg) 36mo	15.0	1.9	Δ wt (kg): 24 - 36mo	2.5	0.9	20.1	7.0
N=666							
Birth length (cm)	49.8	2.1	*Δ ht (cm): 0 - 36mo*	*46*	*3.2*	*92.7*	*8.3*
Height (cm) 6mo	67.2	2.5	Δ ht (cm): 0 - 6mo	17.4	2.1	35.1	5.0
Height (cm) 12mo	75.6	2.7	Δ ht (cm): 6 - 12mo	8.4	1.8	12.6	2.8
Height (cm) 24mo	86.3	3.1	Δ ht (cm): 12 - 24mo	10.8	1.9	14.3	2.5
Height (cm) 36mo	95.8	3.6	Δ ht (cm): 24 - 36mo	9.5	1.6	11.0	1.9

For peer review only

N=682							
Abdominal circum.[2](cm) 0mo	31.5	2.2	*Δ circum. (cm): 0 - 36mo*	*19.8*	*3.1*	*63.3*	*12.3*
Abdominal circum. (cm) 6mo	47.4	3.2	Δ circum. (cm): 0 - 6mo	15.9	3.3	50.9	12.3
Abdominal circum.(cm) 12mo	49.6	3.2	Δ circum. (cm): 6 -12mo	2.2	2.6	4.7	5.7
Abdominal circum. (cm) 24mo	51.9	3.4	Δ circum. (cm): 12 - 24mo	2.3	2.9	4.8	6.0
Abdominal circum.(cm) 36mo	51.3	3.1	Δ circum. (cm): 24 - 36mo	-0.6	2.5	-1.0	4.7
N=645							
Subscapular skinfold (mm) 0mo	5.0	1.0	*Δ subscap[3].(mm): 0 - 36mo*	*1.7*	*1.9*	*37.2*	*42.4*
Subscapular skinfold (mm) 6mo	7.4	1.6	Δ subscap. (mm): 0 - 6mo	2.4	1.7	52.7	41.7
Subscapular skinfold (mm) 12mo	7.2	1.6	Δ subscap. (mm): 6- 12mo	-0.2	1.5	-1.8	19.3
Subscapular skinfold (mm) 24mo	6.6	1.6	Δ subscap. (mm): 12 -24mo	-0.6	1.4	-7.3	19.3
Subscapular skinfold (mm) 36mo	6.7	1.9	Δ subscap. (mm): 24 -36mo	0.1	1.4	3.6	20.1

SD: standard deviation

[1] calculated from previous time interval [2]abdominal circumference [3]subscapular skinfold thickness

For peer review only - http://bmjopen.bmj.com/site/about/guidelines.xhtml

~~Predictors of~~Associations with blood pressure at 36 months

Initially, the four anthropometric measurements were considered separately (Table 3). Each model contained the measurement at birth and the conditional changes in the measure over the four age periods (0-6mo, 6-12mo, 12-24mo, and 24-36mo), along with the confounding factors that contributed significantly to the regression analysis; the slope represents the change in blood pressure (mmHg) per SD change in growth measurement. In all four models, the measurements at birth were not associated with blood pressure, independently of measurements of postnatal growth. The model for abdominal circumference explained more of the variance in blood pressure (~~9.0~~8.8% and 7.7%, respectively, for systolic and diastolic blood pressure) (Figure 1) than the models for the other three measures, with the model for weight coming a close second (6.~~8~~7% and 5.~~3~~5% of the variance explained for systolic and diastolic blood pressure). The change in abdominal circumference ~~in~~closest to the ~~final age period (~~blood pressure measurement, 24-36mo~~)~~, was related most strongly to blood pressure (both systolic and diastolic), but change during the first six months of life was also ~~an important predictor.~~significantly associated with both systolic and diastolic blood pressure. Weight change between birth and 6mo was related to blood pressure at 36mo, but weight change between 12 and 24mo also appeared to influence both systolic and diastolic blood pressure. Neither height nor subscapular skinfold thickness changes were related to blood pressure as strongly as abdominal circumference or weight changes, although for diastolic blood pressure there was a robust association with height change between 12 and 24mo. The effect sizes for the significant associations of body fat distribution and body weight were such that a 1 SDS increase in the measurement between the two ages under consideration was associated with an increase of around 1-2 mmHg in systolic blood pressure and approximately 1 mmHg in diastolic pressure

For peer review only

Table 3. Multiple regression models: associations between conditional gain (z-scores) in body composition measures and blood pressure[1]

-	-	Systolic Pressure[2]					Diastolic Pressure[3]			-
	Adj.R^2	β	95% CI		P-value	Adj.R^2	β	95% CI		P-value
**Model 1 – Weight[4] (N=712)**	0.068	-	-	-	-	.053	-	-	-	-
Birth weight z-score		0.23	-0.41	0.88	0.5		0.04	-0.44	0.52	0.9
Weight: 0-6mo[5]		1.41	0.82	1.99	<0.001		0.82	0.38	1.26	<0.001
Weight: 6-12mo[5]		0.48	-0.11	1.07	0.1		0.34	-0.11	0.78	0.1
Weight: 12-24mo[5]		1.17	0.58	1.76	<0.001		0.73	0.29	1.18	0.001
Weight: 24-36mo[5]		1.03	0.44	1.62	0.001		0.43	-0.02	0.87	0.1
**Model 2 – Length/height[4] (N=666)**	0.029					.038				
Birth length z-score		0.46	-0.29	1.20	0.2		-0.02	-0.56	0.53	0.95
Height: 0-6mo[5]		0.88	0.26	1.50	0.006		0.39	-0.07	0.85	0.1
Height: 6-12mo[5]		0.67	0.04	1.30	0.04		0.27	-0.20	0.73	0.3
Height: 12-24mo[5]		0.73	0.10	1.36	0.02		0.74	0.27	1.21	<0.001
Height: 24-36mo[5]		0.33	-0.30	0.96	0.3		0.18	-0.28	0.65	0.4

For peer review only

**Model 3 – Abdominal circumference[6] (N=682)**	.090					.077				
Birth abdominal circumference z-score		0.27	-0.32	0.87	0.4		-0.29	-0.74	0.16	0.2
Abdominal circumference[5]: 0-6mo		1.56	0.95	2.17	<0.001		1.06	0.60	1.52	<0.001
Abdominal circumerence[5]: 6-12mo		-0.03	-0.63	0.57	0.9		-0.21	-0.67	0.25	0.4
Abdominal circumerence[5]: 12-24mo		0.62	0.02	1.22	0.04		0.42	-0.04	0.87	0.07
Abdominal circumerence[5]: 24-36mo		1.92	1.32	2.51	<0.001		0.97	0.52	1.43	<0.001
**Model 4 – Subscapular skinfold thickness[6] (N=645)**	0.02					.033				
Birth subscapular skinfold		-0.08	-0.74	0.58	0.8		-0.30	-0.79	0.19	0.2
Subscapular skinfold[5]: 0-6mo		0.42	-0.23	1.07	0.2		0.16	-0.33	0.64	0.5
Subscapular skinfold[5]: 6 - 12mo		0.93	0.27	1.58	0.01		0.76	0.28	1.25	0.002
Subscapular skinfold[5]: 12 - 24mo		0.55	-0.10	1.19	0.1		0.05	-0.42	0.53	0.8
Subscapular skinfold[5]: 24 - 36mo	-	0.45	-0.20	1.10	0.2	-	0.25	-0.22	0.73	0.3

CI: confidence interval

[1]residuals derived from regression model with the specified z-score as the independent variable, [2]crying included in the model, [3]crying and maternal educational level included in the model, [4]z-scores derived from percentile curve growth charts UK [15], [5]adjusted for measurements at all preceding time points, [6]z-scores derived internally from Southampton Women's Survey data.

For peer review only

Table 3. Multiple regression models: associations between conditional gain (z-scores) in body composition measures and blood pressure[1,2]

-	-	Systolic Pressure				Diastolic Pressure				-
	Adj.R^2	β	95% CI		P- value	Adj.R^2	β	95% CI		P-value
**Model 1 – Weight[3] (N=684)**	0.067	-	-	-	-	.055	-	-	-	-
Birth weight z-score		0.22	-0.45	0.89	0.5		0.12	-0.38	0.62	0.6
Weight: 0-6mo[4]		1.40	0.76	2.02	<0.001		0.81	0.34	1.27	0.001
Weight: 6-12mo[4]		0.51	-0.09	1.12	0.1		0.36	-0.09	0.82	0.1
Weight: 12-24mo[4]		1.20	0.59	1.81	<0.001		0.76	0.30	1.22	0.001
Weight: 24-36mo[4]		1.07	0.46	1.67	0.001		0.44	-0.01	0.89	0.06
**Model 2 – Length/height[3] (N=649)**	0.027					.038				
Birth length z-score		0.41	-0.36	1.18	0.3		0.08	-0.49	0.65	0.8
Height: 0-6mo[4]		0.91	0.27	1.54	0.005		0.42	-0.05	0.89	0.08
Height: 6-12mo[4]		0.66	0.02	1.30	0.04		0.27	-0.20	0.74	0.3
Height: 12-24mo[4]		0.74	0.09	1.38	0.03		0.72	0.25	1.20	0.003
Height: 24-36mo[4]		0.32	-0.33	0.96	0.3		0.15	-0.32	0.63	0.5

For peer review only

**Model 3 – Abdominal circumference[5] (N=664)**	.088					.077				
Birth abdominal circumference z-score		0.22	-0.40	0.84	0.5		-0.29	-0.76	0.18	0.2
Abdominal circumference[4]: 0-6mo		1.59	0.97	2.21	<0.001		1.04	0.57	1.51	<0.001
Abdominal circumference[4]: 6-12mo		-0.03	-0.64	0.59	0.9		-0.23	-0.70	0.23	0.3
Abdominal circumference[4]: 12-24mo		0.58	-0.03	1.19	0.06		0.46	-0.005	0.92	0.053
Abdominal circumference[4]: 24-36mo		1.84	1.24	2.46	<0.001		1.02	0.56	1.48	<0.001
**Model 4 – Subscapular skinfold thickness[5] (N=630)**	0.018					.035				
Birth subscapular skinfold		-0.17	-0.85	0.52	0.6		-0.25	-0.76	0.26	0.3
Subscapular skinfold[4]: 0-6mo		0.35	-0.33	1.02	0.3		0.09	-0.41	0.59	0.7
Subscapular skinfold[4]: 6 - 12mo		0.99	0.32	1.65	0.004		0.79	0.30	1.28	0.002
Subscapular skinfold[4]: 12 - 24mo		0.52	-0.14	1.17	0.1		0.02	-0.46	0.50	0.9
Subscapular skinfold[4]: 24 - 36mo		0.54	-0.12	1.19	0.1		0.25	-0.24	0.73	0.3

CI: confidence interval

[1]residuals derived from regression model with the specified z-score as the independent variable, [2]all analyses adjusted for crying, maternal education and maternal smoking in late pregnancy, [3]z-scores derived from percentile curve growth charts UK [15], [4]adjusted for measurements at all preceding time points, [5]z-scores derived internally from Southampton Women's Survey data.

For peer review only

The results of the final regression models are presented in Table 4. ~~Both~~In the combined model, both abdominal circumference (0-6mo and 24-36 mo) and weight ~~changes were~~change (12-24mo) remained significantly associated with systolic blood pressure. ~~Not surprisingly, weight gains between birth and 6mo and between 12 and 24mo were associated with abdominal circumference change during these same age periods (r = 0.7 and 0.5 respectively).~~The growth variables contributing to the final models were not highly correlated, with all correlations being less than 0.15. There was considerable variability in the gains of abdominal mass between 24 to 36mo, where 39% of children experienced an increase in abdominal circumference. Abdominal circumference change in the age period leading up to the blood pressure measurement was the key influence on blood pressure, with a 1 SDS change in the circumference being associated with a 1.~~64 mmHg~~66mmHg increase in systolic blood pressure. ~~Early abdominal circumference and weight changes between birth and 6mo were also significantly associated with systolic blood pressure.~~ The final model for diastolic blood pressure was ~~slightly different, though~~ similar, with key ~~predictors were still~~associations being abdominal circumference change in the earliest and latest age periods (0-6mo and 24-36mo) with a 1 ~~SDS~~SD increase in abdominal circumference change associated with approximately 1 mmHg higher diastolic blood pressure. After the abdominal circumference changes were included in the model, changes in weight no longer appeared to influence blood pressure, but height change between 12 and 24mo was retained in the model. The addition of the variables 'ever breast-fed' or 'duration of breast feeding' to the final models did not affect the relationships between blood pressure and body composition measures.

For peer review only

For peer review only - http://bmjopen.bmj.com/site/about/guidelines.xhtml

Table 4. Multivariate regression model of best fit: conditional gain (z-scores)[1] in body size and fat distribution associations with blood pressure at 36 months

**Systolic Pressure**[2]	-				
N=668	Adj.R^2	β	95% CI		P-value
	.098				
Abdominal circumference[3,4]: 0-6mo		0.87	0.02	1.71	0.04
Abdominal circumference[3,4]: 24-36mo		1.64	1.02	2.27	<0.0001
Weight: 0-6mo[3,5]		0.94	0.06	1.82	0.04
Weight: 12-24mo[3,5]		0.77	0.16	1.39	0.01

**Diastolic Pressure**[6]					
N=641	Adj.R^2	β	95% CI		P-value
	.074				
Abdominal circumference[3,4]: 0-6mo		0.98	0.51	1.44	<0.001
Abdominal circumference[3,4]: 24-36mo		0.90	0.43	1.38	<0.001
Height: 12-24mo[3,5]		0.60	0.12	1.07	.01

CI: confidence interval

[1]residuals derived from regression model with the specified z-score as the independent variable, [2]crying included in the model, [3]adjusted for all the preceding time intervals (0-6mo, 6-12mo, 12-24mo, 24-36mo), [4]Z-scores based on sample population data from Southampton Women's Survey, [5]z-scores derived from percentile curve growth charts UK [15], [6]crying and maternal educational level included in the model

For peer review only

Table 4. Multivariate regression model of best fit: conditional gain (z-scores) in body size and fat distribution associations with blood pressure at 36 months[2]

**Systolic Pressure** N=650	Adj.R^2	β	95% CI		P-value
	.094				
Abdominal circumference[3,4]: 0-6mo		1.52	0.07	1.71	0.04
Abdominal circumference[3,4]: 24-36mo		1.66	0.94	2.27	<0.0001
Weight: 12-24mo[3,5]		0.79	0.16	1.39	0.01

**Diastolic Pressure** N=625	Adj.R^2	β	95% CI		P-value
	.076				
Abdominal circumference[3,4]: 0-6mo		0.95	0.48	1.42	<0.001
Abdominal circumference[3,4]: 24-36mo		0.94	0.46	1.43	<0.001
Height: 12-24mo[3,5]		0.60	0.12	1.08	0.02

CI: confidence interval

[1]residuals derived from regression model with the specified z-score as the independent variable, [2]all analyses adjusted for crying, maternal education and maternal smoking in late pregnancy, [3]adjusted for all the preceding time intervals (0-6mo, 6-12mo, 12-24mo, 24-36mo), [4]Z-scores based on sample population data from Southampton Women's Survey, [5]z-scores derived from percentile curve growth charts UK [15]

Formatted: Font: 12 pt, Not Bold, Font color: Auto

Formatted: Font: 12 pt, Font color: Auto

For peer review only - http://bmjopen.bmj.com/site/about/guidelines.xhtml

DISCUSSION

Formatted: Normal

Main findings

Our data indicate that conditional gains in abdominal circumference between birth and 6 months, as well as in the 12 months prior to blood pressure measurement, ~~are significant predictors of~~were associated with both systolic and diastolic blood pressure at 36 months of age. Changes in weight were also associated with blood pressure, particularly systolic pressure, but to a lesser extent. ~~A~~ In the multivariate regression model, a one-~~SDS z-score~~SD gain in abdominal circumference z-score between 24 and 36mo was associated with a 1.~~64~~66 mmHg increase in systolic blood and a one-~~SDS~~SD increase between birth and 6mo with ~~0.87 mmHg~~1.56mmHg.

There was considerable variability in the gains of abdominal mass at the time interval most predictive of blood pressure (24 to 36mo), with 39% of children experiencing an increase, suggesting the impact of environmental factors. It is possible that dietary intake and/or physical activity levels during this early period could alter abdominal circumference gain early in life, or it may be related to differences in growth trajectories,[16] which predict later obesity. Given that abdominal circumference in the majority of children decreases between the ages of 24 and 36mo, increases seen in this time period might be an early marker for problems in later life, particularly if the effect of abdominal circumference change on blood pressure amplifies with age.

It is of note that subscapular skinfold thickness change, an indicator of subcutaneous fat deposition, had much weaker associations with blood pressure than abdominal circumference, which is an indicator of abdominal or visceral fat deposition. The finding that abdominal circumference gain between 0-6mo was more strongly associated with blood pressure at age 36mo than gains during 6-12mo and 12-24mo could reflect the proposed critical period for childhood adiposity in the first two months of life,[17] or could reflect gains in adiposity associated with an adverse intrauterine environment in late gestation.

For peer review only

Strengths and weaknesses

As summarised earlier, a strength of this study is the large sample size drawn from the general population. A post-hoc power calculation (n=650, with SD of 8mmHg for systolic pressure) indicated there was 80% power to detect a regression coefficient of 0.88mmHg per 1SD of an independent variable at 5% level of significance. Our study was thus sufficiently large to detect effect sizes of clinical relevance. In addition, we have assessed conditional growth, accounting for measurements at previous ages (thus ensuring independence of growth summaries between age periods), and have adjusted for relevant confounding factors.

Few studies have collected such detailed anthropometric measurements with the methodological rigor to provide indicators of fat distribution in the early postnatal period, encompassing the transition from milk-only to a mixed diet at the age of 36 months.

Formatted: Indent: First line: 0.5"

It is clear from the data that the strongest association with blood pressure from all the anthropometric measurements is the gain in abdominal circumference; however, we acknowledge that when building a multivariate regression model of best fit, there is an element of chance as to which variables are included, even when they are not highly correlated.

A limitation of the study is the inability to measure blood pressure in all children in the cohort, although there do not appear to be noteworthy differences between those who were measured and the remainder.

For peer review only

For peer review only - http://bmjopen.bmj.com/site/about/guidelines.xhtml

Comparison with other studies

Central adiposity and a large waist circumference have been associated with higher blood pressure in adults, but there are few reports for children. In one study, children with accelerated weight gain in the first two years of life were fatter and had more central fat distribution at five years of age than other children.[6] However, the relationship of central fat deposition specifically to blood pressure, which is only one component of the metabolic syndrome, is not clear in children. Rapid weight gain during infancy (0–6mo) but not during early childhood (3–6 years) was associated with a higher metabolic risk score at age 17 years, but the association between waist circumference and blood pressure was not statistically significant in that study.[18] Waist-for-height ratio in school children has not been found to confer additional discriminative power to BMI in some studies,[19, 20] but recently weight and weight-for-height changes through infancy and childhood have been associated with blood pressure at age 10 years[21] and at age 9.1 and 15.5 years.[22] These studies did not consider the periods 0-6mo and 6-12mo separately, nor examine change in abdominal circumference. However, similar to both reports, we found that weight changes at ages closest to those at which the blood pressure measurement was made were among the strongest predictors.most strongly associated.

There is considerable debate as to whether there are specific age-periods early in life during which weight gain and/or fat deposition are more influential in predicting cardiovascular risk. Our findings are similar to a report that weight gain between 24 and 36mo was positively associated with systolic blood pressure.[23] The mean blood pressure values in our study of 94/58 mmHg are similar to those reported (92/58 mmHg),[23] as are the correlations between systolic blood pressure and current weight and height at 36mo. Similarly, we also did not find an inverse association between birth weight and blood pressure. However, in contrast to the other findings that blood pressure was related to weight change between 24 and 36mo but not earlier, we found that conditional gains in body weight between all age periods were positively associated with systolic blood pressure and a similar trend for diastolic pressure, except for the period between 6 to 12mo. The

For peer review only - http://bmjopen.bmj.com/site/about/guidelines.xhtml

differences in results may be related to our adjustment for all previous time intervals when we determined conditional growth.

Our data support a developmental contribution to the origin of elevated blood pressure in childhood, and therefore potentially in later adulthood. Indeed, we have previously demonstrated that higher fetal liver blood flow is strongly correlated with greater fat mass at birth and at 4 years, indicating that influences on body composition operate very early in life.[24] Our data show that changes in abdominal circumference during the first six months and in the third year of life are associated with systolic and diastolic blood pressure. This suggests that the tracking of blood pressure, evident by later childhood, might be a consequence of under-supply of conditionally essential nutrients associated with elevated fetal liver blood flow and prioritisation of fat deposition in the infant.

It is not clear whether postnatal interventions would be effective in limiting growth in abdominal circumference, and any interventions to limit abdominal circumference gains would likely also reduce the rate of growth. However, there is accumulating evidence that accelerated early growth has long-term adverse physiological effects in later life, increasing cardiovascular risk factors, including high blood pressure.[25] Promotion of lifestyle practices, such as exclusive breast feeding up to 6 months, later introduction of solid foods and maximising opportunities for safe physical activity, could contribute to a reduction in accelerated growth early in life and be one pathway that results in a reduction in cardiovascular disease later in life.

Conclusion

We have demonstrated that although conditional gains in body weight and height during specific age periods are associated with systolic and diastolic blood pressure, as previously recognised, conditional gains in abdominal circumference (associated with central fat deposition) arehave the strongest predictors ofassociation with blood pressure at 36 months of age. This is especially true for the first 6 months of life and

For peer review only - http://bmjopen.bmj.com/site/about/guidelines.xhtml

between 24 and 36 months (i.e., the year prior to blood pressure measurement). Therefore, central deposition of fat in early childhood, indicated by abdominal circumference, may contribute to an increased risk of developing hypertension later in life. Follow-up blood pressure measurements for the study group may enable confirmation of this at a later date.

For peer review only

For peer review only - http://bmjopen.bmj.com/site/about/guidelines.xhtml

Figure 1

Association between change in abdominal circumference (residuals derived from regressing the z-score at each specific age on the z-score at all preceding ages, adjusted for crying and maternal educational attainment and smoking during pregnancy) as the independent variable and blood pressure at 36 months (mmHg). Z-scores are based on data from the Southampton Women's Survey, adjusted for gender, current age and gestational age.

For peer review only

For peer review only - http://bmjopen.bmj.com/site/about/guidelines.xhtml

Acknowledgements

The Southampton Women's Survey Study Group includes Patsy Coakley, Vanessa Cox, Julia Hammond, Tina Horsfall and the Research Nurses who collected and processed the data for the Survey. The authors are very grateful to the families who participated in the research.

Competing Interests

None

Funding

This work was supported by grants from the Medical Research Council, British Heart Foundation, Arthritis Research UK, National Institute for Health Research (NIHR) Southampton Biomedical Research Centre, University of Southampton and University Hospital Southampton NHS Foundation Trust, and NIHR Musculoskeletal Biomedical Research Unit, University of Oxford. The work was also financially supported in part by the Commission of the European Community, specifically the RTD Programme "Quality of Life and Management of Living Resources", within the 7th Framework Programme, research grant no. FP7/2007-13 (Early Nutrition Project). This manuscript does not necessarily reflect the views of the funders and in no way anticipates the future policy in this area. KMG is supported by the National Institute for Health Research through the NIHR Southampton Biomedical Research Centre.

Author contributions

Caryl Nowson conceptualised the study, performed the analysis, contributed to the interpretation and drafted the initial manuscript. Sarah Crozier assisted with the analysis and contributed to the interpretation. Siân Robinson contributed to interpretation. Keith Godfrey, Wendy Lawrence, Catherine Law and Cyrus Cooper

For peer review only

reviewed and revised the manuscript. Hazel Inskip conceptualised the study and designed the analysis plan, performed analysis, contributed to the interpretation and revised the manuscript. All authors approved the final manuscript as submitted.

Data sharing

No additional data available.

For peer review only

REFERENCES

1 Ioannou C, Javaid MK, Mahon P, *et al.* The effect of maternal vitamin D concentration on fetal bone. *J Clin Endocrinol Metab* 2012;97:E2070-7.

2 Law CM, Shiell AW, Newsome CA, *et al.* Fetal, infant, and childhood growth and adult blood pressure: a longitudinal study from birth to 22 years of age. *Circulation* 2002;105:1088-92.

3 Noakes PS, Vlachava M, Kremmyda LS, *et al.* Increased intake of oily fish in pregnancy: effects on neonatal immune responses and on clinical outcomes in infants at 6 mo. *Am J Clin Nutr* 2012;95:395-404.

4 Huxley RR, Shiell AW, Law CM. The role of size at birth and postnatal catch-up growth in determining systolic blood pressure: a systematic review of the literature. *J Hypertens* 2000;18:815-31.

5 Barker DJ, Osmond C, Golding J, *et al.* Growth in utero, blood pressure in childhood and adult life, and mortality from cardiovascular disease. *BMJ* 1989;298:564-7.

6 Ong KK, Ahmed ML, Emmett PM, *et al.* Association between postnatal catch-up growth and obesity in childhood: prospective cohort study. *BMJ* 2000;320:967-71.

7 Forsen T, Nissinen A, Tuomilehto J, *et al.* Growth in childhood and blood pressure in Finnish children. *J Hum Hypertens* 1998;12:397-402.

8 Jarvelin MR, Sovio U, King V, *et al.* Early life factors and blood pressure at age 31 years in the 1966 northern Finland birth cohort. *Hypertension* 2004;44:838-46.

9 Ben-Shlomo Y, McCarthy A, Hughes R, *et al.* Immediate postnatal growth is associated with blood pressure in young adulthood: the Barry Caerphilly Growth Study. *Hypertension* 2008;52:638-44.

10 Fabricius-Bjerre S, Jensen RB, Faerch K, *et al.* Impact of birth weight and early infant weight gain on insulin resistance and associated cardiovascular risk factors in adolescence. *PLoS One* 2011;6:e20595.

For peer review only

For peer review only - http://bmjopen.bmj.com/site/about/guidelines.xhtml

11 Bai J, Abdul-Rahman MF, Rifkin-Graboi A, *et al.* Population Differences in Brain Morphology and Microstructure among Chinese, Malay, and Indian Neonates. *PLoS One* 2012;7:e47816.

12 O'Tierney PF, Lewis RM, McWeeney SK, *et al.* Immune response gene profiles in the term placenta depend upon maternal muscle mass. *Reprod Sci* 2012;19:1041-56.

13 Inskip HM, Godfrey KM, Robinson SM, *et al.* Cohort profile: The Southampton Women's Survey. *Int J Epidemiol* 2006;35:42-8.

14 Whincup PH, Bruce NG, Cook DG, *et al.* The Dinamap 1846SX automated blood pressure recorder: comparison with the Hawksley random zero sphygmomanometer under field conditions. *J Epidemiol Community Health* 1992;46:164-9.

15 Cole TJ, Freeman JV, Preece MA. Body mass index reference curves for the UK, 1990. *Arch Dis Child* 1995;73:25-9.

16 Rolland-Cachera MF, Deheeger M, Maillot M, *et al.* Early adiposity rebound: causes and consequences for obesity in children and adults. *Int J Obes (Lond)* 2006;30 Suppl 4:S11-7.

17 Stettler N, Tershakovec AM, Zemel BS, *et al.* Early risk factors for increased adiposity: a cohort study of African American subjects followed from birth to young adulthood. *Am J Clin Nutr* 2000;72:378-83.

18 Qiu A, Fortier MV, Bai J, *et al.* Morphology and microstructure of subcortical structures at birth: A large-scale Asian neonatal neuroimaging study. *Neuroimage* 2012;65C:315-23.

19 Harvey NC, Mahon PA, Kim M, *et al.* Intrauterine growth and postnatal skeletal development: findings from the Southampton Women's Survey. *Paediatr Perinat Epidemiol* 2012;26:34-44.

20 Harvey N, Dhanwal D, Robinson S, *et al.* Does maternal long chain polyunsaturated fatty acid status in pregnancy influence the bone health of children? The Southampton Women's Survey. *Osteoporos Int* 2012;23:2359-67.

For peer review only

For peer review only - http://bmjopen.bmj.com/site/about/guidelines.xhtml

21 Jones A, Charakida M, Falaschetti E, *et al.* Adipose and height growth through childhood and blood pressure status in a large prospective cohort study. *Hypertension* 2012;59:919-25.

22 Chiolero A, Paradis G, Bovet P. Which period of growth is determinant for blood pressure? *Hypertension* 2012;60:e10; author reply e1.

23 Hindmarsh PC, Bryan S, Geary MP, *et al.* Effects of current size, postnatal growth, and birth size on blood pressure in early childhood. *Pediatrics* 2010;126:e1507-13.

24 Godfrey KM, Haugen G, Kiserud T, *et al.* Fetal liver blood flow distribution: role in human developmental strategy to prioritize fat deposition versus brain development. *PLoS One* 2012;7:e41759.

25 Singhal S, A. Early growth and later atherosclerosis. *World Rev Nutr Diet* 2013;106:162-7.

26 Wells JC, Chomtho S, Fewtrell MS. Programming of body composition by early growth and nutrition. *Proc Nutr Soc* 2007;66:423-34.

For peer review only

For peer review only - http://bmjopen.bmj.com/site/about/guidelines.xhtml

Figure 1

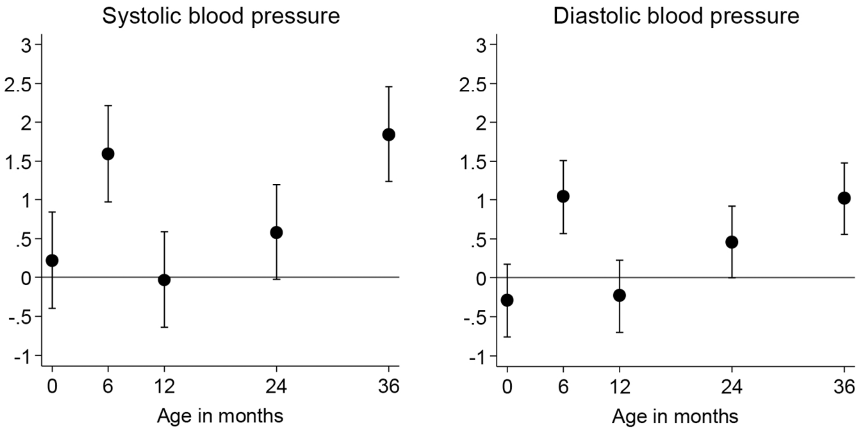

Association between change in abdominal circumference (residuals derived from regressing the z-score at each specific age on the z-score at all preceding ages, adjusted for crying and maternal educational attainment and smoking during pregnancy) as the independent variable and blood pressure at 36 months (mmHg). Z-scores are based on data from the Southampton Women's Survey, adjusted for gender, current age and gestational age.
152x84mm (300 x 300 DPI)

STROBE Statement—Checklist of items that should be included in reports of ***cohort studies***

	Item No	Recommendation	Addressed in article?
**Title and abstract**	1	(*a*) Indicate the study's design with a commonly used term in the title or the abstract	Yes
		(*b*) Provide in the abstract an informative and balanced summary of what was done and what was found	Yes
**Introduction**			
Background/rationale	2	Explain the scientific background and rationale for the investigation being reported	Yes
Objectives	3	State specific objectives, including any prespecified hypotheses	Yes
**Methods**			
Study design	4	Present key elements of study design early in the paper	Yes
Setting	5	Describe the setting, locations, and relevant dates, including periods of recruitment, exposure, follow-up, and data collection	Yes
Participants	6	(*a*) Give the eligibility criteria, and the sources and methods of selection of participants. Describe methods of follow-up	Yes
		(*b*) For matched studies, give matching criteria and number of exposed and unexposed	N/A
Variables	7	Clearly define all outcomes, exposures, predictors, potential confounders, and effect modifiers. Give diagnostic criteria, if applicable	Yes
Data sources/ measurement	8*	For each variable of interest, give sources of data and details of methods of assessment (measurement). Describe comparability of assessment methods if there is more than one group	Yes
Bias	9	Describe any efforts to address potential sources of bias	Yes
Study size	10	Explain how the study size was arrived at	Yes
Quantitative variables	11	Explain how quantitative variables were handled in the analyses. If applicable, describe which groupings were chosen and why	Yes
Statistical methods	12	(*a*) Describe all statistical methods, including those used to control for confounding	Yes
		(*b*) Describe any methods used to examine subgroups and interactions	Yes
		(*c*) Explain how missing data were addressed	Yes
		(*d*) If applicable, explain how loss to follow-up was addressed	Yes
		(*e*) Describe any sensitivity analyses	N/A
**Results**			
Participants	13*	(a) Report numbers of individuals at each stage of study—eg numbers potentially eligible, examined for eligibility, confirmed eligible, included in the study, completing follow-up, and analysed	Yes
		(b) Give reasons for non-participation at each stage	Yes
		(c) Consider use of a flow diagram	Not thought

For peer review only - http://bmjopen.bmj.com/site/about/guidelines.xhtml

			necessary
Descriptive data	14*	(a) Give characteristics of study participants (eg demographic, clinical, social) and information on exposures and potential confounders	Yes
		(b) Indicate number of participants with missing data for each variable of interest	Yes
		(c) Summarise follow-up time (eg, average and total amount)	Yes
Outcome data	15*	Report numbers of outcome events or summary measures over time	Yes
Main results	16	(*a*) Give unadjusted estimates and, if applicable, confounder-adjusted estimates and their precision (eg, 95% confidence interval). Make clear which confounders were adjusted for and why they were included	Yes
		(*b*) Report category boundaries when continuous variables were categorized	Yes
		(*c*) If relevant, consider translating estimates of relative risk into absolute risk for a meaningful time period	N/A
Other analyses	17	Report other analyses done—eg analyses of subgroups and interactions, and sensitivity analyses	Yes
**Discussion**			
Key results	18	Summarise key results with reference to study objectives	Yes
Limitations	19	Discuss limitations of the study, taking into account sources of potential bias or imprecision. Discuss both direction and magnitude of any potential bias	Yes
Interpretation	20	Give a cautious overall interpretation of results considering objectives, limitations, multiplicity of analyses, results from similar studies, and other relevant evidence	Yes
Generalisability	21	Discuss the generalisability (external validity) of the study results	Yes
**Other information**			
Funding	22	Give the source of funding and the role of the funders for the present study and, if applicable, for the original study on which the present article is based	Yes

*Give information separately for exposed and unexposed groups.

Note: An Explanation and Elaboration article discusses each checklist item and gives methodological background and published examples of transparent reporting. The STROBE checklist is best used in conjunction with this article (freely available on the Web sites of PLoS Medicine at http://www.plosmedicine.org/, Annals of Internal Medicine at http://www.annals.org/, and Epidemiology at http://www.epidem.com/). Information on the STROBE Initiative is available at http://www.strobe-statement.org.

For peer review only
